# Establishing a consensus on the clinical assessment of Hippocratic temperaments in the French-speaking naturopathic community: a Delphi study

**DOI:** 10.1186/s12906-026-05353-y

**Published:** 2026-03-29

**Authors:** Ludivine Colas, Anne Gimalac, Carine Bucher, Susanti Chandra, Nora Eberli, Amie Steel

**Affiliations:** 1 Navi - Research in Integrative Health, Lausanne, Switzerland; 2 Australian Research Consortium in Complementary and Integrative Medicine, School of Public Health, Faculty of Health, University of Technology Sydney, Ultimo, Australia

**Keywords:** Naturopathy, Delphi, Hippocratic temperament, Humoral medicine, Holistic health, Traditional medicine, Clinical assessment

## Abstract

**Background:**

The concept of Hippocratic temperaments is widely used in Europe for naturopathic clinical assessment. However, there is currently no consensus model for understanding the application of Hippocratic temperaments in this context, although this would seem to be an essential step towards the scientific evaluation of this traditional humoral theory. The aim of this study is to reach a consensus on how French-speaking naturopaths currently define the concept of temperament, apply humoral theory and characterize the four Hippocratic temperaments in naturopathic assessment.

**Methods:**

A Delphi study was conducted over a 19-month period in four rounds with 66 expert panelists. Consensus was defined as a median value of 75% or greater agreement with the relevant statement.

**Results:**

Consensus was met with 217 statements describing the concept of temperament and the four temperaments in terms of psycho-emotional dimension, behavior, physiology, morphology and susceptibility to disease.

**Conclusions:**

This data highlights the relevance of this model for French-speaking practitioners, while also illustrating the major potential of such a holistic approach to health prevention, individualized care and interdisciplinary work. Although further research phases are still required, it is our intention that these results will contribute to creating a scientific assessment tool.

**Supplementary Information:**

The online version contains supplementary material available at 10.1186/s12906-026-05353-y.

## Background

Naturopathic medicine is a holistic healthcare system which recognizes the body’s innate healing capacity, emphasizes disease prevention and health promotion and blends traditional philosophies and practices with scientific advances and current research [1, 2]. Currently, over 110,000 naturopaths and naturopathic doctors practice in over 108 countries [3] and deliver person-centered healthcare [4] using a breadth of natural treatment modalities and practices in accordance with their core naturopathic principles, philosophies and theories [5].

Humorism is one of these theories with deep historical roots in Europe [6]. Records of the humoral theory relating to medicine can be found in the Corpus Hippocraticum (460–377 B.C.), a collection of early ancient Greek medical works [6–8]. According to humorism, the universe comprises four elements: fire, water, earth, and air [6, 9]. These elements of the macrocosm (universe) are also found on a smaller scale as humors composing each human being (microcosm) [6, 9]. Conforming to an equilibrium model, a balance of the four humors results in health, whereas a marked qualitative or quantitative imbalance results in disease [7–9]. However, because this balance is different in each person, explaining physiologic interindividual differences in psychology, behavior, physiology and morphology, it is crucial to determine the patient’s normal humoral condition before assessing the changes in humors that caused the disease [7]. In this context, the individual physiologic humoral condition of a person is described with the assistance of the Hippocratic temperament theory [7]. This theory describes four temperaments: phlegmatic, sanguine, choleric and melancholic, and is spread integratively throughout the world, like in Middle Eastern humoral medicine [10] or North American and European herbalism [11–13].

In Europe, humorism retains a contemporary resonance, where it is used as a central theory by naturopathic practitioners [6]. It seems to take pride of place in naturopathic clinical assessment, as the concept of traditional humoral theories is found in 95% of naturopathic courses (compared to 74% internationally) [14], and its application to naturopathic assessment through traditional humoral diagnosis is taught in 80% of naturopathic education programs (against 60% worldwide) [14].

During naturopathic clinical assessment, practitioners must skillfully combine their traditional prisms (such as humorism) with standard conventional health assessment techniques [2], continually bridging traditional knowledge and biomedical science. This is where the Hippocratic theory of temperaments is included in a person-centered assessment process, which seeks to determine the factors contributing to the patient’s state of health, their symptoms and conditions [15].

Moreover, this complex process must always be consistent with the philosophical foundations of practice, which guide all aspects of naturopathic care: on one side holism, complementary philosophy to reductionism [16], considering the body as a complex adaptive system that exists as a unified whole [5, 17], and on the other side vitalism, which sees every human being animated by an innate intelligence that gives the body a natural ability to balance itself, and gives a certain logic to symptoms and illnesses [5, 6].

Modern implementation of traditional knowledge like Hippocratic temperaments requires continued engagement with critical evaluation, to determine which knowledge and practices align authentically with the philosophical roots of the traditional medicine system and are appropriate for the contemporary context [18]. Until today, scientific modeling of the temperaments has been dominated by Eysenck's operationalization of Galen's typology [19–23], which is applied within various fields of psychology. However, it is based on the assessment of two personality traits only: extraversion (tendency to enjoy social events and interaction) and neuroticism (tendency to experience negative emotions) [19]. This twentieth century model has been integrated into scientifically validated tests commonly used, such as the Cloninger’s Temperament and Character Inventory [24]. As there is currently no assessment model taking into account all relevant dimensions of Hippocratic temperaments for naturopathic practice, Cloninger's model is used by certain research teams in traditional medicine [25] seeking to establish connections between their practice and the European humoral model.

In an effort to support naturopathic medicine with robust methodologies, the aim of this study is thus to define a consensus that describes how French-speaking naturopaths currently define the concept of a temperament, apply humoral theory, and characterize the four Hippocratic temperaments for naturopathic assessment. The purpose is to have a consensus-based model to understand the application of this specific humoral theory in naturopathic clinical assessment.

## Materials and methods

### Design

Four-round Modified-Delphi study.

This consensus-building study was designed and conducted by using a four-round Modified-Delphi technique. This iterative survey method allows purposive sampling of recognized experts across geographically diverse locations, while providing heterogeneity, anonymity, equality, controlled feedback, and aggregation of participant views [26–29].

The four rounds of questionnaires were originally created for the study (see Appendix 1). Round one defined the concept of temperament, laying the foundations for understanding the theory and completing the literary references used to create the item bank. Rounds two to four were then used to explore the assessment characteristics of each temperament taking into account the foundations of round one and informed by existing literature. A closing criterion was defined by setting the number of questionnaire rounds at four, based on similar naturopathic research [30].

### Panelist selection

The study used purposive and snowball sampling to select expert panelists. Panelists had to meet the following inclusion criteria: (1) Fluent comprehension of written and oral French, (2) Current clinician, author, educator or researcher, (3) Familiar with applying the Hippocratic temperaments to teaching, research or clinical practice. Students in naturopathy without any practice experience were excluded. Diversity in gender, type of affiliation, number of years of experience and country of activity was considered during panelist selection.

### Panelist recruitment

Recruitment was conducted via email invitation using a standardized script combined with a Google form. This form was used for registration and to ensure that all participants met the inclusion criteria. Firstly, the invitation was sent to organizations that were asked to diffuse it to their members: World Naturopathic Federation (WNF), French Organization of Natural Medicine and Health Education (OMNES), French Federation of Naturopathy (FENA), as well as major professional associations and schools in France, Switzerland, Belgium, Luxemburg, Canada (Quebec). Secondly, the invitation was sent to experts who were asked to personally participate and/or forward the invitation to other experts (snowball sampling approach). Informed consent to participate was obtained from all of the participants, at the time of initial recruitment, and at the start of each round. A link to the online survey information sheet (see Appendix 2) was provided at each of these stages, with the information that submission of the online questionnaire counts as validation of informed consent.

### Data collection

The research was conducted over a 19-month period and the four Delphi rounds were carried out between March and June 2023.

### Baseline instrument development

A literature review of 23 books, scientific publications and professional content was conducted between June and August 2022. These sources were selected for their high degree of precision when describing each Hippocratic temperament (see Appendix 3). Texts in English or German were translated by bilingual research team members so that all literature extracts relating to the description of Hippocratic temperaments were reported in French. Data from the review was extracted into a Microsoft Excel spreadsheet and classified according to five dimensions: (1) Morphology—Physical appearance (general appearance, body, face, hand, tongue, etc.); (2) Physiology—Physiological functioning of the body in the absence of disease (sleep, appetite, weight gain, coldness, sweating, etc.); (3) Psycho-emotional—Mental functions and feelings; (4) Behavior—Behaviors and reactions in terms of actions; (5) Susceptibility to diseases—Susceptibility to specific and recurrent pathologies.

Following this matrix structure of four temperaments by five dimensions, a large bank of items was created for building up the Delphi rounds. It included items related to the Hippocratic temperament concept in naturopathy, strictly extracted from the literature without editing, as well as items related to temperament characteristics, created by reformulating the most commonly cited characteristics in the selected literature. For each citation of a characteristic by an author (e.g., introversion for lymphatic temperament), the item was scored with one rating point in the bank.

### Instrument administration

The Delphi rounds were conducted using the Qualtrics software. Each participant received an invitation email outlining the study procedures, the completion deadline, and a weblink to the online questionnaire for each round. Panelists were given three to four weeks to complete each round, with a reminder sent one week before the round’s closure. The questionnaire would take 20 to 30 min to complete. At the start of each round, panelists' demographics (e.g. gender, nationality) and professional characteristics (e.g. institutions, years of experience) were collected, to capture and understand their engagement and enable finer result analysis of each round. From round one to round four, participants responded to the questionnaire using a five-point Likert scale (1. Strongly disagree, 2. Disagree, 3. Neutral, 4. Agree, 5. Strongly agree).

#### Round 1

**Fig. 1:**
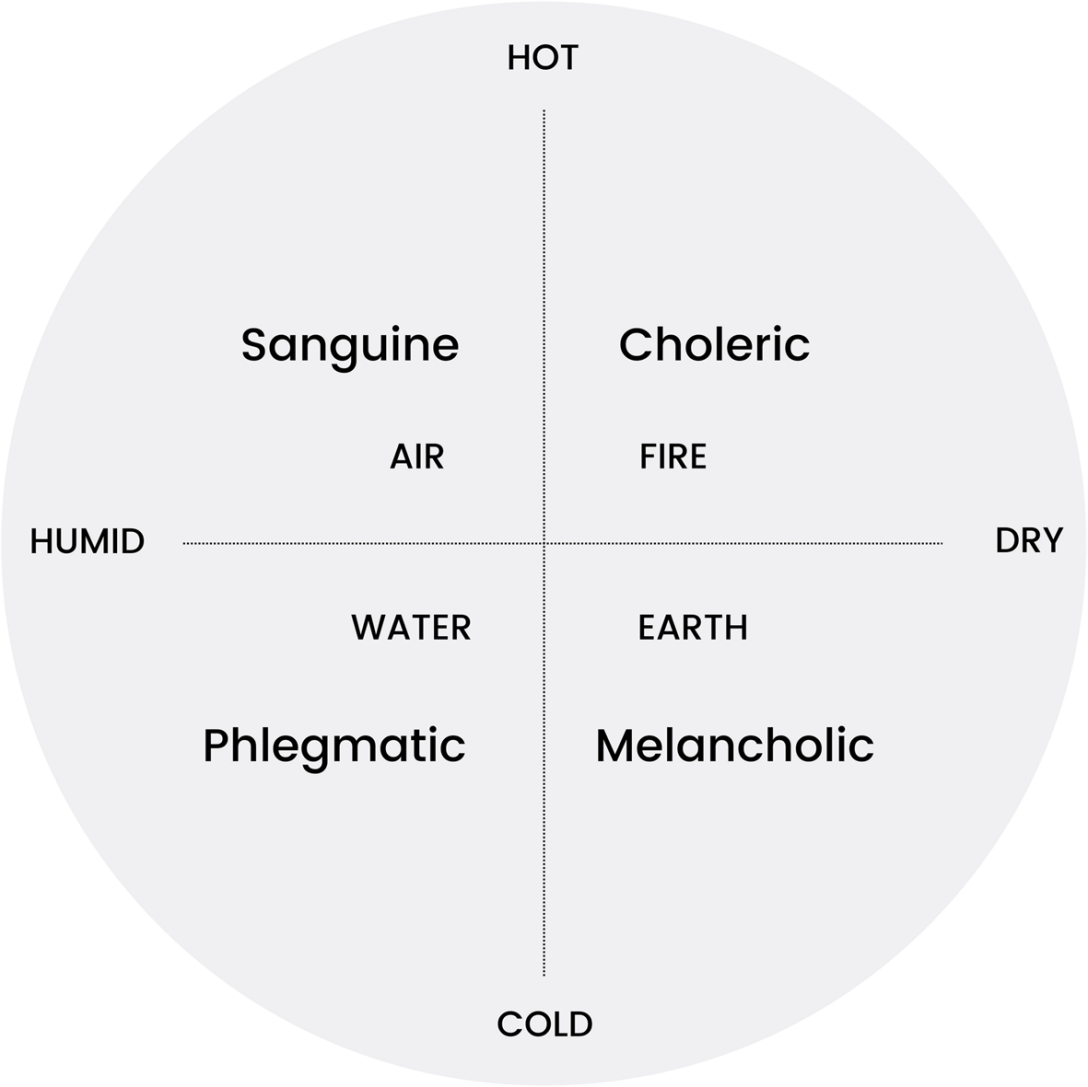
Temperament quadrant

Round one focused on defining temperament and the fundamentals of Hippocrates’ theory with items from the literature. Participants were first provided with a list of all references used to inform the items included in the instrument (see Appendix 1 and 3). They were invited to suggest additional references they considered important sources of knowledge on Hippocratic temperaments. Participants' perspectives on the concept of temperament were then investigated. They had to score both affirmations and a graphical representation (Fig. 1). The quadrant was an opportunity for the panelists to give their level of agreement with the qualities associated with the four temperaments (cold, hot, dry, humid), as well as their dynamics (e.g. expansion attributed to humid, retraction attributed to dry) and associated elements (earth, air, water, fire). Finally, panelists were asked to rank the five temperament dimensions by order of importance, and could formulate one or more additional dimensions they considered important.

#### Round 2

In order to create round two, additional references identified by participants were reviewed, leading to an enrichment of the item bank. Panelists’ ranking of the importance of temperament dimensions were also used to adapt the order of dimension presentation. Finally, the responses meeting consensus criteria were analyzed to create an initial definition of the naturopathic concept of temperament that was shared with the panelists for further development. The round two questionnaire was divided into seven sections: Panelists demographics (Part A), Concept of temperament (Part B), Psycho-emotional dimension (Part C), Behavioral dimension (Part D), Physiological dimension (Part E), Morphological dimension (Part F) and Susceptibility to diseases dimension (Part G). Except from Part A and B, each section proposed one to nine affirmations characterizing each Hippocratic temperament.

#### Rounds 3 and 4

The final two rounds further refined the items based on panelist input. At the beginning of rounds three and four, participants were provided the number of items that were accepted and rejected at the previous round, without disclosing the specific items. At the end of round three, some items that did not meet the consensus or exclusion criteria were re-worded or split into several new items for greater clarity, and included in round four. No new items were added in round four, at the end of which the termination criteria was reached.

### Data management and analyses

Data analysis was undertaken between rounds and occurred from July to November 2023. A *priori* consensus for automatically including an item was defined using percentile score when ≥ 75% of participants responded ‘4. Agree’ or ‘5. Strongly agree’ (Score of 4 or 5 at the 25th percentile). Items attracting < 50% agreement (Score of 3. Neutral, 2. Disagree or 1. Strongly disagree” at the 50th percentile) were automatically excluded. Any remaining items were forwarded to the subsequent round and from round three, revised through discussion amongst the research team taking into account panelists' comments. Any item not reaching 75% of agreement at the last round was excluded. Items that failed to reach the consensus or exclusion criteria were reinjected in the next round.

The mean scores of the five temperament dimensions were compared using one-way analysis of variance (ANOVA). Post-hoc pairwise comparisons were performed using Tukey’s HSD test to identify statistically significant differences between dimensions. A p-value < 0.05 was considered statistically significant.

Templates and tables were created on Google sheets for initial quantitative and qualitative analysis to collate, summarize, interpret and integrate data. Data were then exported from Qualtrics as spreadsheets and analyzed using Stata 17.1 software to determine consensus.

## Results

### Panelists

A total of 66 French-speaking naturopaths registered at the time of initial recruitment, and the average participation in the four rounds was 38.75 panelists (median = 39), i.e. 58.7% of registrants.

The initial sample included participants working from four main countries: France (73.5%), Switzerland (16.2%), Belgium (8.8%) and Canada (2.9%). Some panelists reported working in more than one of these countries. Panelists reported practicing mainly as clinicians (61.78%) and teachers (24.44%). Most of them worked in a private practice (62.69%) and in school or vocational training center (38.2%). In terms of experience with the Hippocratic theory of temperament, a majority of participants (28.42%) reported having less than five years experience, while 20% had more than ten years. Finally, women represented three-quarters of the participants registered, although their commitment was less consistent over the four rounds compared to men (Men participation at initial recruitment: 14,7%; four rounds mean: 23,9% vs Women participation at initial recruitment: 85,3%; four rounds mean: 76,15%). For further detail on participant characteristics and involvement see Table 1.

**Table 1:** Panelist demographics

Characteristic		Result					
**Research steps**		**Time of initial recruitment**	**Round 1**	**Round 2**	**Round 3**	**Round 4**	**Average of the four rounds**
Panelists		66	39	39	39	38	38,75
Gender	Female	85.30%	76.92%	74.36%	74.36%	78.95%	76.15%
	Male	14.70%	23.08%	25.64%	25.64%	21.05%	23.85%
Country of practice	France	73.50%	51.11%	60.00%	58.14%	59.52%	57.19%
	Switzerland	16.20%	22.22%	24.44%	20.93%	19.05%	21.66%
	Belgium	8.80%	11.11%	4.44%	9.30%	9.52%	8.59%
	Canada	5.90%	11.11%	4.44%	6.98%	7.14%	7.42%
	Other	10%	4.44%	6.67%	4.65%	4.76%	5.13%
Practice related to temperament	Naturopathic clinician	98.50%	59.38%	56.72%	70.37%	60.66%	61.78%
	Researcher	1.50%	0.00%	1.49%	0.00%	0.00%	0.37%
	Teacher	32.40%	26.56%	20.90%	24.07%	26.23%	24.44%
	Author	8.80%	4.69%	4.48%	1.85%	6.56%	4.40%
	Practitioner in another discipline (phytotherapy, dietetics, etc.)	17.60%	4.69%	10.45%	3.70%	6.56%	6.35%
	Other	2.90%	4.69%	5.97%	0.00%	0.00%	2.67%
Practice institution	Private practice	89.70%	59.68%	60.66%	67.27%	63.16%	62.69%
	School or vocational training center	38.20%	30.65%	29.51%	25.45%	28.07%	28.42%
	University	0%	1.61%	0.00%	1.82%	0.00%	0.86%
	Laboratory	4.40%	1.61%	3.28%	0.00%	3.51%	2.10%
	Research center	0.00%	0.00%	0.00%	0.00%	0.00%	0.00%
	Other	14.50%	6.45%	6.56%	4.45%	5.26%	5.68%
Years of experience	< 5	n/a	46.15%	43.59%	51.28%	44.74%	46.44%
	5 ≤ x ≤ 10	n/a	30.77%	35.90%	30.77%	34.21%	32.91%
	10 >	n/a	23.08%	20.51%	17.95%	21.05%	20.65%

### General consensus results

**Fig. 2:**
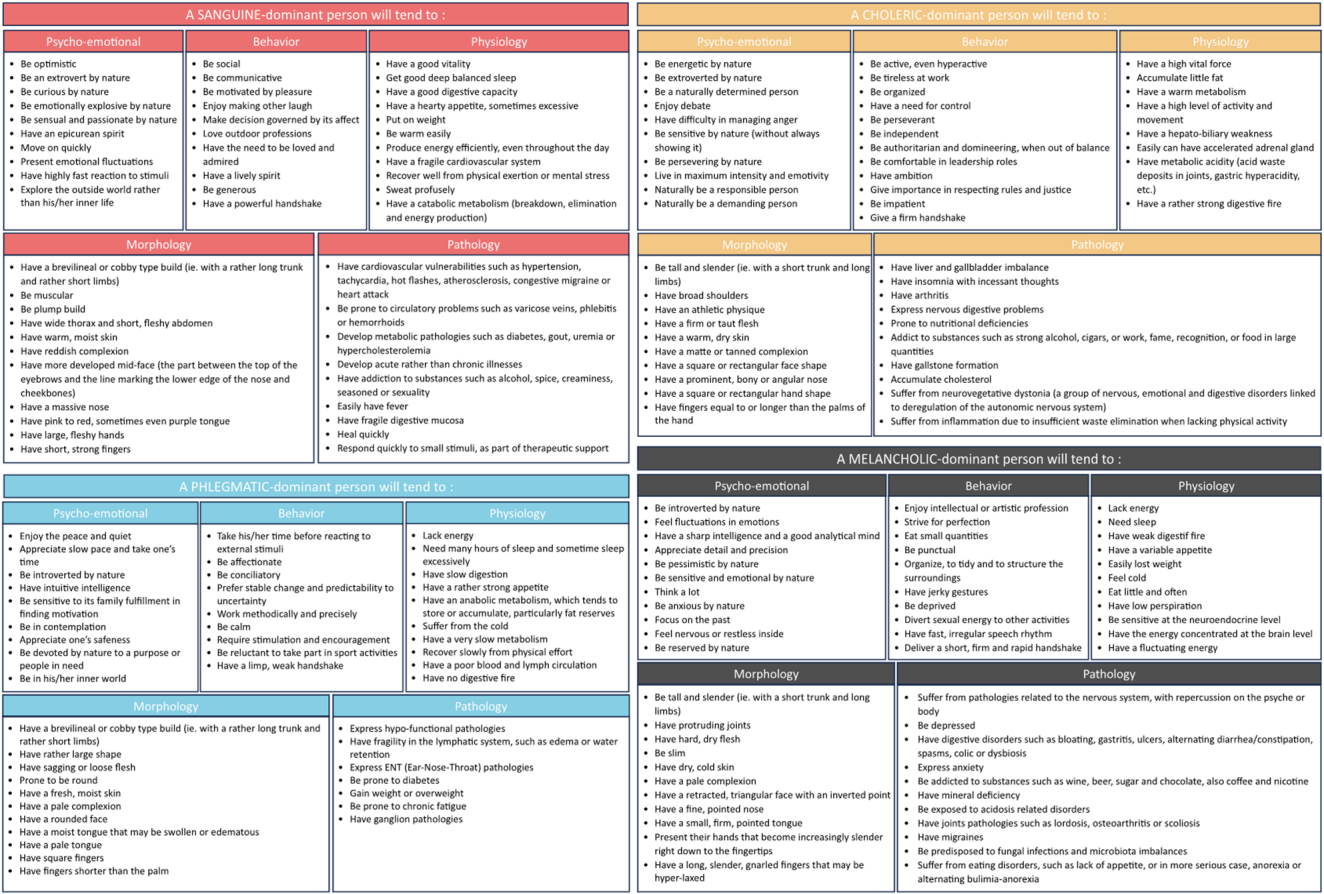
217 items categorized by temperament and dimension

Of the 274 survey items, 217 reached consensus for inclusion (Table 2, Fig. 2) while the remaining were excluded (Table 3) by reaching the exclusion criteria or not reaching the consensus criteria at the end of the fourth and last round (closing criteria). All the data presented in the following detailed paragraphs can be found in the two above-mentioned tables.

**Table 2:** 217-Item consensus about Hippocrates theory of temperaments in naturopathic clinical assessment

	Dimension	Temperament	Item	Round consensus achieved*
1	Temperament's notion		In practice, we describe four main temperaments: Lymphatic (or phlegmatic or pituitary), sanguine, choleric and melancholic	1
2			The four temperaments are our uniquely human way of expressing the balance of four forces that govern all thing according to the ancient way of understanding character and health. These four forces are the humors, which mix and move within us and affect every aspect of our lives	1
3			A person's temperament is the combination of the humors that compose them	1
4			The temperament is one of the expressions of the terrain	1
5			The temperament is the activity of vital forces as it manifests in each individual	2
6			Actual individuals most often belong to several types, sometimes with a marked dominance. It can be said that an individual is never a pure type	1
7			We are always imbued with a fundamental temperament that characterizes us, but it can fade in favor of one or more temperaments that emerge over the course of our life	1
8			Temperament is inherently innate, but it also stems from the acquired	2
9			Temperament is far from being fixed, it is a reflexion of life, it is an ongoing dynamic that is sometimes excessively or insufficiently expressed	1
10			Temperament in itself has not pathological significance. However, the dominating humor tends to be a potential pathogen is stimuli can no longer be compensated	1
11			Temperament is not definitive, it evolves over the course of life in accordance with lived situations	1
12			Temperament expresses individual unity that emcompasses physiological, morphological and psychological traits	1
13			In the presented diagram, do you agree with the distribution of the elements (earth/water/fire/air) associated with each temperament?	2
14			In the presented diagram, do you agree with the distribution of the qualities (hot/dry/cold/moist) associated with each temperament?	1
15			Do you agree with the fact that the moisture principal is asociated with dilation (governs phlegmatic and sanguine profiles)?	1
16			Do you agree with the fact that the dryness principal is asociated with constriction (governs choleric and melancholic profiles)?	1
17	Psycho-emotional	Phlegmatic	Enjoy the peace and quiet	2
18			Appreciate a slow pace and taking one's time	2
19			Be introverted by nature	3
20			Have intuitive intelligence	2
21			Be sensitive to their family fulfillment to find motivation	3
22			Be comtemplative	3
23			Value safety	3
24			Be devoted to causes or people in need by nature	3
25			Be in his/her inner world	3
26		Sanguine	Be optimistic	2
27			Be extraverted by nature	2
28			Be curious by nature	2
29			Be emotionally explosive by nature	2
30			Be sensual and passionate by nature	2
31			Have an epicurian spirit	2
32			Move on quickly	3
33			Present emotional fluctuations	3
34			React quickly to stimuli	3
35			Explore the outside world rather than his/her inner life	3
36		Choleric	Be energetic by nature	2
37			Be extroverted by nature	3
38			Be determined by nature	3
39			Enjoy debates	3
40			Have difficulty managing anger	3
41			Be sensitive by nature (without always showing it)	3
42			Be perseverent by nature	3
43			Live at maximum intensity and emotionality	3
44			Be responsible by nature	3
45			Be demanding by nature	3
46		Melancholic	Be introverted by nature	2
47			Feel emotional fluctuations	2
48			Have a sharp intelligence and a good analytical mind	2
49			Appreciate details and precision	2
50			Be pessimistic by nature	3
51			Be sensitive and emotional by nature	2
52			Reflect a lot	3
53			Be anxious by nature	3
54			Focus on the past	4
55			Feel internal nervousness or restlessness	3
56			Be reserved by nature	3
57	Behavior	Phlegmatic	Take his/her time before reacting to external stimuli	2
58			Be affectionate	2
59			Be conciliatory	2
60			Prefer stability over change and predictability over uncertainty	2
61			Work methodically and precisely	3
62			Be calm	3
63			Require stimulation and encouragement	3
64			Be reluctant to take part in sports	3
65			Have a limp, weak handshake	3
66		Sanguine	Be social	2
67			Be communicative	2
68			Be motivated by pleasure	2
69			Enjoy making others laugh	2
70			Make decisions based on their affect	2
71			Love outdoor professions	3
72			Have the need to be loved and admired	3
73			Have a lively spirit	3
74			Be generous	3
75			Have a strong handshake	3
76		Choleric	Be active, even hyperactive	2
77			Be tireless at work	2
78			Be organized	2
79			Have a need for control	2
80			Be perseverant	2
81			Be independent	2
82			Be authoritarian and domineering, when out of balance	3
83			Be comfortable in leadership roles	3
84			Have ambition	3
85			Give importance to following rules and justice	3
86			Be impatient	3
87			Give a firm handshake	3
88		Melancholic	Enjoy artistic or intellectual professions	2
89			Strive for perfection	2
90			Eat small quantities	2
91			Be punctual	2
92			Organize, tidy and structure their environment	2
93			Have jerky gestures	3
94			Be deprived	3
95			Divert sexual energy to other activities	4
96			Have a quick and irregular rhythm in their speech	3
97			Deliver a short, firm and rapid handshake	3
98	Physiology	Phlegmatic	Lack energy	3
99			Need many hours of sleep and sometime sleep excessively	2
100			Have slow digestion	2
101			Have a rather strong appetite	3
102			Have an anabolic metabolism, which tends to store or accumulate, particularly fat reserves	2
103			Suffer from the cold	3
104			Have a very slow metabolism	3
105			Recover slowly from physical effort	3
106			Have a poor blood and lymph circulation	3
107			Have no digestive fire	3
108		Sanguine	Have a good vitality	2
109			Have a good deep balanced sleep	2
110			Have a good digestive capacity	2
111			Have a hearty appetite, sometimes excessive	2
112			Put on weight	2
113			Be warm easily	2
114			Produce energy efficiently, even throughout the day	3
115			Have a fragile cardiovascular system	3
116			Recover well from physical exertion or mental stress	3
117			Sweat profusely	3
118			Have a catabolic metabolism (breakdown, elimination and energy production)	3
119		Choleric	Have a strong vital force	2
120			Accumulate little fat	2
121			Have a warm metabolism	2
122			Have a high level of activity and movement	3
123			Have a hepato-biliary weakness	3
124			Easily present with accelerated adrenal glands	3
125			Have metabolic acidity (acid waste deposits in joints, gastric hyperacidity, etc.)	3
126			Have a rather strong digestive fire	3
127		Melancholic	Lack energy	3
128			Need sleep	2
129			Have weak digestif fire	2
130			Have a variable appetite	2
131			Lose weight easily	2
132			Feel cold	2
133			Eat little and often	3
134			Sweat little	3
135			Be sensitive at the neuroendocrine level	3
136			Have their energic concentrated at the level of the brain	3
137			Have a fluctuating energy	3
138	Morphology	Phlegmatic	Have a brevilineal or cobby type build (ie. with a rather long trunk and rather short limbs)	3
139			Have rather large shape	2
140			Have sagging or loose flesh	2
141			Be prone to be round	2
142			Have a fresh, moist skin	2
143			Have a pale complexion	2
144			Have a rounded face	3
145			Have a moist tongue that may be swollen or edematous	3
146			Have a pale tongue	4
147			Have square fingers	4
148			Have fingers shorter than the palm	3
149		Sanguine	Have a brevilineal or cobby type build (ie. with a rather long trunk and rather short limbs)	2
150			Be muscular	2
151			Have a plump build	2
152			Have a wide thorax and a short and fleshy abdomen	2
153			Have warm, moist skin	2
154			Have reddish complexion	2
155			Have more developed mid-face (the part between the top of the eyebrows and the line marking the lower edge of the nose and cheekbones)	3
156			Have a massive nose	3
157			Have pink to red, sometimes even purple tongue	3
158			Have large, fleshy hands	3
159			Have short, strong fingers	3
160		Choleric	Be tall and slender (ie. with a short trunk and long limbs)	2
161			Have broad shoulders	2
162			Have an athletic physique	2
163			Have a firm or taut flesh	2
164			Have a warm, dry skin	2
165			Have a matte or tanned complexion	2
166			Have a square or rectangular face shape	3
167			Have a prominent, bony or angular nose	3
168			Have a square or rectangular hand shape	3
169			Have fingers equal to or longer than the palms of the hand	3
170		Melancholic	Be tall and slender (ie. with a short trunk and long limbs)	2
171			Have protruding joints	2
172			Have hard, dry flesh	2
173			Be slim	2
174			Have dry, cold skin	2
175			Have a pale complexion	2
176			Have a retracted, triangular face with an inverted point	3
177			Have a fine, pointed nose	3
178			Have a small, firm, pointed tongue	3
179			Have hands that are increasignly slender from palm to fingertips	3
180			Have long and slender fingers with enlarged joints that may be hypermobile	3
181	Susceptibility to diseases	Phlegmatic	Express hypo-functional pathologies	2
182			Have fragility in the lymphatic system, such as edema or water retention	2
183			Express ENT (Ear-Nose-Throat) pathologies	3
184			Be prone to diabetes	2
185			Gain weight or be overweight	3
186			Be prone to chronic fatigue	3
187			Have lymph node pathologies	3
188		Sanguine	Have cardiovascular vulnerabilities such as hypertension, tachycardia, hot flashes, atherosclerosis, congestive migraine or heart attack	2
189			Be prone to circulatory problems such as varicose veins, phlebitis or hemorrhoids	2
190			Develop metabolic pathologies such as diabetes, gout, uremia or hypercholesterolemia	2
191			Develop acute rather than chronic illnesses	2
192			Have addiction to substances such as alcohol, spice, creaminess, seasoned or sexuality	2
193			Easily have fever	3
194			Have frail digestive mucosa	4
195			Heal quickly	3
196			Respond quickly to small stimuli, as part of therapeutic support	3
197		Choleric	Have liver and gallbladder imbalance	2
198			Have insomnia with incessant thoughts	2
199			Have arthritis	2
200			Express nervous digestive problems	2
201			Prone to nutritional deficiencies	2
202			Have addictions to substances such as spirits, cigars, or work, fame, recognition or food in excess	2
203			Have gallstone formation	3
204			Accumulate cholesterol	3
205			Suffer from neurovegetative dystonia (a group of nervous, emotional and digestive disorders linked to deregulation of the autonomic nervous system)	4
206			Suffer from inflammation due to insufficient waste elimination when lacking physical activity	3
207		Melancholic	Suffer from pathologies related to the nervous system, with repercussion on the psyche or body	2
208			Be depressed	2
209			Have digestive disorders such as bloating, gastritis, ulcers, alternating diarrhea/constipation, spasms, colic or dysbiosis	2
210			Express anxiety	2
211			Be predisposed to fungal infections and microbiota imbalances	3
212			Have adictions to subtances such as wine, beer, sugar and chocolate, and also coffee and nicotine	3
213			Have mineral deficiencies	3
214			Be exposed to pathologies relating to acidosis	3
215			Have joints pathologies such as lordosis, osteoarthritis or scoliosis	3
216			Have migraines	3
217			Suffer from eating disorders, such as lack of appetite, or in more serious case, anorexia or alternating bulimia-anorexia	3

^*^Consensus was defined using percentile score when ≥ 75% of participants responded ‘4.Agree’ or ‘5.Strongly agree’ (Score of 4 or 5 at 25th percentile) to an item

**Table 3:** Excluded items

	Dimension	Temperament	Item	Consensus round
1	Temperament's notion		Temperament is that aspect of our personalities that is genetically based, inborn, there from birth or even before	1
2			If they [temperaments] are on an uncertain path, their relative dynamics show more of a mixture of the four constitutional values distributed individually, than a true evolutionary or involutionary shift from one temperament to another	1
3			Temperament is the main setting of the constitution	1
4			The four temperaments are the basic constitutional bodymind types of Greek Medicine	2
5			Extraversion (E, the tendency to enjoy social events and interaction) and Neuroticism (N, the tendency to experience negative emotions) […] reflect Hippocrates’ four temperaments	1
6			The four fundamental tendencies or temperaments (sanguine, phlegmatic, choleric, melancholic) correspond to the development of the four primordial instincts (breathing, eating, moving, thinking) which trigger a preference in the search for certain vital stimulants (air, food, exercise, psychic excitement) according to individual predominances	2
7	Psycho-emotional	Phlegmatic	Be emotionally stable by nature	4
8			Be creative	4
9			Appreciate solitude	4
10			Appreciate individual conversations	4
11		Sanguine	Be creative by nature	4
12			Avoid conflict	3
13		Choleric	Have fluctuating emotions (moodswings)	3
14			Control their emotions	4
15		Melancholic	Have great capacity for change	3
16	Behavior	Phlegmatic	Have a great capacity for adaptation	4
17			Be methodical in their work	4
18			Be precise in their work	4
19			Be perseverent or enduring	3
20			Be present with things, events and people, thanks to their capacity to be anchored	4
21		Sanguine	Lack perseverence	4
22			Be easily influenced by certain people	4
		Choleric	–-	–-
23		Melancholic	Have a soft and timid handshake	2
24			Do little physical activity	4
25	Physiology	Phlegmatic	Have great endurance	3
26			Have touch as their dominant sense	4
27		Sanguine	Have smell as their dominant sense	4
28			Have taste as their dominant sense	4
29		Choleric	Have restorative sleep	3
30			Digest quickly	4
31			Digest well	4
32			Have a large appetite	2
33			Use sight as their main sense	4
34		Melancholic	Use hearing as their main sense	3
35	Morphology	Phlegmatic	Have a small nose	4
36		Sanguine	Have a large tongue that is swollen on occasion	3
37		Choleric	Have a red tongue	4
38			Have a tongue with red dots on the surface	4
39			Have a dry tongue	4
40			Have a tongue with cracks or fissures	4
41		Melancholic	Have a pale tongue	4
42			Have a blueish tongue	4
43	Susceptibility to diseases	Phlegmatic	Have cutaneous issues	2
44			Have addictions to substances such as cigarettes	4
45			Have addictions to substances such as beer	4
46			Be addicted to helping others	4
47			Suffer from constipation	3
48			Be depressed	4
49			Respond to intense and long lasting stimuli, as part of a therapeutic accompaniement	4
50		Sanguine	Have respiratory frailties	4
51			Develop hepatic insufficiency	4
52			Have frail respiratory mucosa	4
53			Have frail genitourinary mucosa	4
54			Have mild childhood allergies, which become excessive weight gain by adulthood	4
55		Choleric	Have poor immunity	3
56			Develop severe symptoms at the onset of disease from lack of energy stores (vitality) or moisture	4
57		Melancholic	Develop serious or degenerative diseases, including auto-immuno, neuro-endocrine or cancers	3

For round 4 (final round), exclusion criteria applied to items not reaching 75% agreement (Score of 3.Neutral, 2.Disagree or 1.Strongly disagree” at 25th percentile)

^*^From round 1 to 3, exclusion criteria was defined for items attracting < 50% agreement (Score of 3.Neutral, 2.Disagree or 1.Strongly disagree” at 50th percentile)

The response rate remained very stable between rounds as 39 participants completed the survey for round one, two and three (59% response rate), and 38 panelists completed round four of the survey (57,6% response rate).

**Fig. 3:**
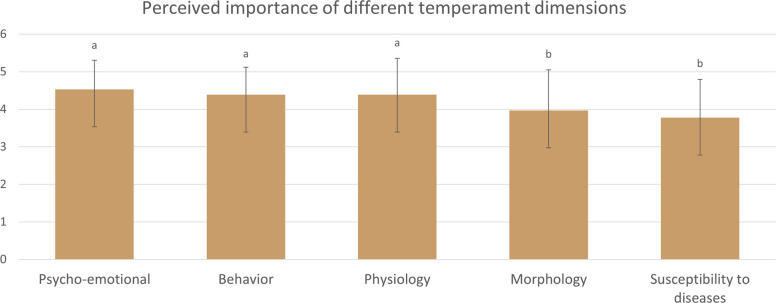
Perceived importance of different temperament dimensions (1-least important, 5-most important). Different letters above bars indicate statistically significant differences between temperament dimensions (Tukey’s HSD, *p* < 0.05)

At the end of the first round, the panelists ranked the dimensions of Hippocratic temperaments in order of importance for the assessment of patients. Psycho-emotional dimension was considered as the most important and susceptibility to diseases as the least important (Fig. 3). A one-way ANOVA revealed a statistically significant difference in the perceived importance scores among the five temperament dimensions (*p* < 0.05). Post-hoc Tukey’s HSD analysis showed that the dimensions Psycho-emotional, Behavior, and Physiology did not differ significantly from each other (*p* > 0.05), but all three had significantly higher scores compared to Morphology and Susceptibility to diseases (*p* < 0.05). No significant difference was found between Morphology and Susceptibility to diseases (*p* > 0.05).

### Round 1 results

The list of 23 literary references presented to the experts led to the suggestion of three new bodies of work. As two of them were not available (books out of print), only one was considered and used for enriching the bank of items characterizing Hippocratic temperaments.

Out of the 18 proposals from the literature without reformulation, ten reached consensus for inclusion, four reached consensus for exclusion, and four were forwarded to round two for re-rating. The four proposals relating to the Temperament Quadrant (Fig. 1) led to agreement on the associated qualities (cold, hot, dry, humid) as well as their dynamics (expansion-retraction). The item concerning the associated elements (earth, air, water, fire) was forwarded to the subsequent round.

### Round 2 results

In addition to the five first-round items to be re-examined, 120 new items focusing on temperament evaluation criteria were presented. They were explored dimension by dimension, according to the order given by the first-round results on the importance of these dimensions (Fig. 3), from the most important to the least important. Of the 125 survey items, 98 reached consensus for inclusion, five reached consensus for exclusion, and 22 were forwarded to the next round for re-rating.

### Round 3 results

The statements included the 22 forwarded items from round two and 120 new proposals, all of them focused on the evaluation criteria for each temperament. Of the 142 survey items, 100 reached consensus for inclusion, eleven reached consensus for exclusion. Of the remaining 31 items, ten were selected on the basis of panelists' comments, and reworded or split into several items for greater clarity. This resulted in the production of 41 items (instead of 31) to be re-injected into round four. No new items were added for this last round, as explained in the previous section.

### Round 4 results

Of the 43 survey items, six reached consensus for inclusion. Five reached consensus for exclusion and the remaining 32 items were excluded, as specified in the exclusion criteria for this final round. As such, termination criteria were achieved for these remaining items, and the survey was completed.

## Discussion

This study presents the results of the first attempt to apply modern scientific rigor to the clinical assessment of the patient’s Hippocratic profile. However, a consensus does not mean that the correct answer, opinion or judgment has been found [31]. Yet, with 66 panelists registered, a median participation of 39 panelists, and 217 items reaching consensus, this broadly achieved consensus-based characterization has succeeded in depicting how a traditional humoral theory is applied in the contemporary practice of French-speaking naturopathy. Moreover, the participant rate shows that the temperament theory might still be important to French-speaking naturopaths.

A consensus on the current definition of a temperament, the application of Hippocrates theory and the characterization of the four Hippocratic temperaments is the basis for the creation of a rigorous assessment tool such as a scientifically validated questionnaire. Assessing a patient's Hippocratic temperament allows evaluation of inter-individual differences among people from a humoral point of view. This identification and description of the individual condition is the basis for individualizing and optimizing naturopathic interventions. When regarding other traditional medicine systems, such as traditional Korean medicine [32–35], Unani-Tibb medicine [10, 36] or traditional Chinese medicine [37–39], the importance of temperament-based, individually optimized interventions have been scientifically proven. However, this is not yet the case for European humoral medicine. As such, this study addresses a critical gap in the field and gives naturopathic scientists the opportunity to provide a unique European perspective on humorism and health assessment.

The consensus identified through this study presents dynamics associated with temperament, such as expansion-retraction, and highlights five dimensions that structure 201 individual characteristics. Overall this illustrates an interesting insight into the complexity and holistic nature of naturopathic assessment, while at the same time questioning the relevance of Eysenck's model to naturopathic practice [40]. The assessment across several interconnected dimensions is in line with the philosophy of holism previously described [5, 16, 17], and suggests that humoral theories applied to naturopathic assessment calls for investigation through the linking of different dimensions of the human being. This interconnected approach to health assessment bridges both with other traditional medicines [5, 10, 41, 42] that share this operating basis, and with certain emerging biomedical disciplines such as psychoneuroimmunoendocrinology [43–45], genomic medicine [46, 47] or integrative oncology [48, 49], that consider different dimensions or systems of the human being and describe their interactions. The Hippocratic humoral assessment model seems thus conducive to interdisciplinary work, as it is both relevant and meaningful across multiple medicine models.

Consensus was also reached on the order of importance given by practitioners to the five evaluated dimensions. For clinical assessment, the psycho-emotional dimension was rated as the most important while susceptibility to diseases was considered the least important. This finding may be interpreted as a shift from conventional medical practice, where symptom assessment typically receives the greatest attention. However, without compromising the importance of symptoms, we can recontextualize that this humoral assessment model is designed not only for cases where disease is already present, but also for individuals who do not yet show any symptoms [7]. As disease prevention and the attainment of optimal health are primary objectives of naturopathic practice [1, 6], this weighting of assessment dimensions could render the Hippocratic temperament model a valuable preventive tool. Again, this may align with other traditional medicine systems that have scientifically formalized their humoral assessment tool to study their impact on disease prevention [35, 50].

Placing thoughts and emotions at the forefront of assessment may also reflect the founding principles of practice “Treat the Cause” (tolle causam) [6]. The latter emphasizes the need to look beyond symptoms to understand the deeper origins and reasons for illness susceptibility. This principle is intrinsically linked to the preventive approach, as addressing the root causes of disease inherently involves preventing its onset. To effectively treat and prevent symptoms, it is important to understand not only each person’s physiology and pathology, but also their emotional states, perceptions of health and disease [6], lifestyle habits, and health history [8]. French-speaking panelists may apply this principle consciously or unconsciously, developing a complementary assessment to conventional medicine, with great potential for disease prevention and accompaniment of certain pathologies such as functional or chronic disorders.

### Strengths and limitations

One of the great strengths of this study is that it has clarified the dimensions and characteristics of assessments that currently seem relevant to naturopaths for evaluating a person’s Hippocratic temperament. This enables practitioners to refer to a framework that complements the highly diversified professional and mainstream literature, which is more finely tuned but also more contrary in terms of opinions. As a next step, it seems meaningful to create a scientific tool to assess Hippocratic temperaments and then to test individualized naturopathic intervention measures according to these temperaments in order to determine whether this allows achievement of improved health outcomes.

A limitation is that this work only reflects a few French speaking countries, which implies the need for additional research to include more French-speaking experts from more countries, as well as to develop transferability to countries with other languages. Contacted experts from Africa, Eastern Mediterranean Region, or smaller European countries like Luxembourg, despite the presence of naturopathic schools in these countries, did not volunteer to participate in this study. This may indicate that Hippocratic theory is not used in these settings. Therefore, the consensus reflects a particular regional lens and may not be generalizable to other naturopathic contexts where humoral traditions have evolved differently.

Another limitation is that this study could have benefited from more integration of German-language temperament literature [51–56], which is very rich but also requires a great deal of translation and analysis.

A third limitation may be the relatively small sample size of registered experts. However, as the Delphi method prioritizes participant expertise over sample size, and given the limited number of experts available in French-speaking countries, this constraint reflects the specialized nature of the field. While a larger panel would have been desirable, consensus was broadly achieved among participants, suggesting that the inclusion of additional experts might not have substantially changed the findings.

## Conclusion

This study aimed to identify a consensus among French-speaking naturopathic practitioners on the use of Hippocratic temperament theory in naturopathic assessment. Panel members reached consensus on 217 affirmations that are relevant to describing the temperament concept and each profile in terms of psycho-emotional dimension, behavior, physiology, morphology and susceptibility to disease. The identification of these five interconnected dimensions and their many characteristics illustrates the centrality of the holistic and preventive approach to naturopathic clinical assessment, while attempting to turn the page on the application of Eysenck's model to talk about temperaments in the field of traditional medicine. Further research is required to explore how such a humoral theory, which embodies the holistic approach, may be a key to fostering interdisciplinary work, both with other biomedical disciplines and between traditional medicines around the world.

As a unique European perspective on humorism and health assessment, Hippocratic temperament theory offers a foundational consensus to inform the development of individualized assessment tools for future clinical application. In order to pursue this goal, further research is needed to create a scientifically validated questionnaire to reliably determine Hippocratic temperaments in naturopathic clinical practice. 

## Supplementary Information

Supplementary Material 1. Appendix 1. Original online survey questionnaire. English language version of the four original questionnaire rounds created in French for the study, based on the literature review and the results of each previous round.

Supplementary Material 2. Appendix 2. Online survey information sheet. Information sheet detailing the research and its conditions, sent to participants at the time of initial recruitment and presented to the UTS ethics committee.

Supplementary Material 3. Appendix 3. Selected texts from the literature review. A literature review of 23 books, scientific publications and professional content was carried out between June and August 2022. The full list of these references was provided to participants in the first survey round, informing them of the literary sources used to construct the questionnaires and inviting them to suggest additional references they considered important sources of knowledge on Hippocratic temperaments.

## Data Availability

The datasets generated and analyzed during the current study are available at [http://repository-a.hippocrate-science.epsn.ch/].

## Abbreviations

FENA: French Federation of Naturopathy
OMNES: French Organization of Natural Medicine and Health Education
WNF: World Naturopathic Federation
