# Supplementary material for: Establishing a consensus on the clinical assessment of Hippocratic temperaments in the French-speaking naturopathic community: a Delphi study
Source: BMC Complement Med Ther. 2026 Mar 29;26:173. doi: 10.1186/s12906-026-05353-y (PMC13151160; doi:10.1186/s12906-026-05353-y)
Supplement: Supplementary file 1 — Supplementary Material 1. Appendix 1. Original online survey questionnaire. English language version of the four original questionnaire rounds created in French for the study, based on the literature review and the results of each previous round. [file 12906_2026_5353_MOESM1_ESM.docx]

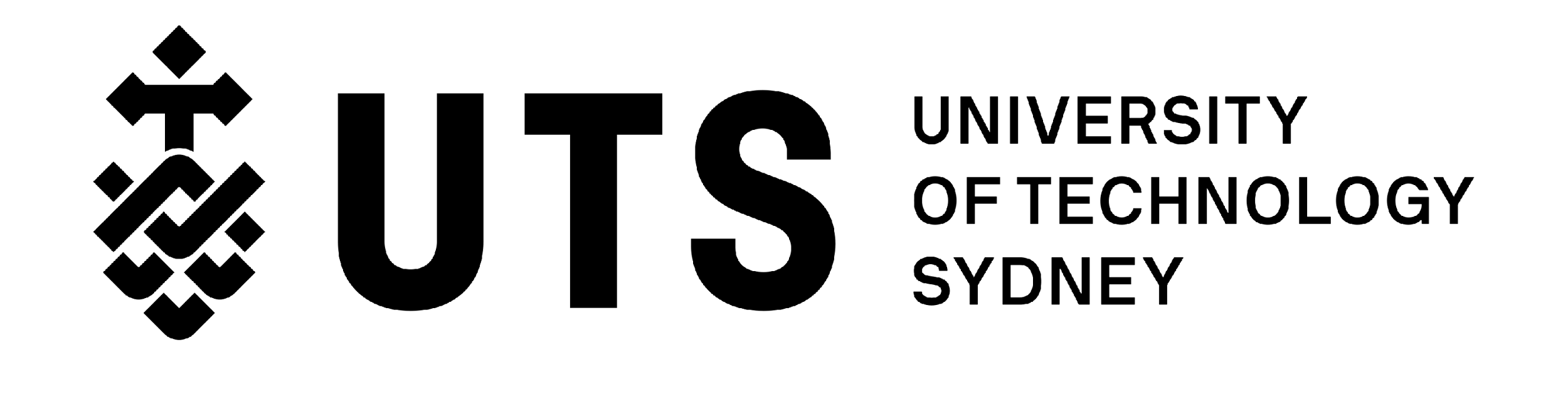


**Appendix 1: Original online survey questionnaire**

## **HIPPOCRATE & SCIENCE PROJECT**

**Hippocratic profiles and temperaments evaluation tool**

**for scientific research and clinical practice in naturopathy**

**PHASE A: CONSENSUS ON TEMPERAMENT THEORY AND ITS APPLICATION IN NATUROPATHY**

**[ETH22-7865 ETHICS COMMISSION APPROVAL].**


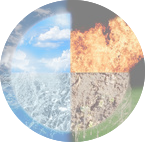


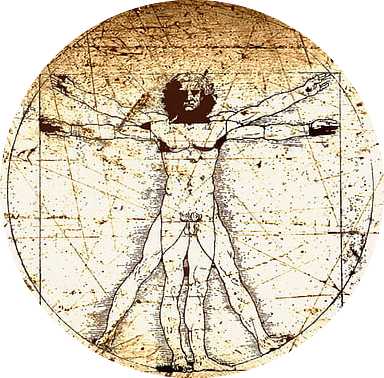


FIRST ROUND OF DELPHI QUESTIONNAIRE (1/4)

**1.1. INTRODUCTION**

## **Rationale for this research project**

## The naturopathic discipline is developing innovative and integrative research approaches in today's scientific world. One of its challenges is to transcribe and restore the founding philosophies and theories of the field of practice, in order to remain as close as possible to clinical reality and better explain the choice of care interventions.

## To achieve this, new, proven scientific tools can be developed to transcribe the analytical grids relevant to the assessment of the patient/client's *terrain*. The Hippocratic temperament theory is a classic naturopathic basis for clinical analysis, derived from traditional Greek medicine, and is used to assess the patient's *terrain*. The phlegmatic, sanguine, choleric or melancholic dominances identified guide the choice of lifestyle advice, cures or natural remedies.

**Objective of your participation in this questionnaire**

The aim of the questionnaire you are about to complete is to find a consensus that reflects the reality of the practice and teaching of this theory in the French-speaking naturopathic community. To this end, you will be asked to make a number of propositions (such as affirmations) taken from the literature. You must position yourself from the point of view of your practical and/or theoretical experience to specify whether they reflect your understanding and practice of Hippocratic theory, as it has been taken up and worked on in the field of naturopathy today. Please note that after each round, we take your answers and comments into account to build the questionnaire for the next round and thus refine the consensus.

**Participant information sheet**

By clicking on this link, you can consult the participant information sheet sent to you during recruitment. We remind you that submitting the questionnaire online is an indication of your consent.

## **1.2. ABOUT YOU**

Before we start, we need to gather some information about your practice again, to ensure that we collect a wide variety of perspectives on the subject, as well as to be able to relate these perspectives to the study results.

## **1.2.1. Which genre do you identify with?**

◻ Female

◻ Male

◻ Other

**1.2.2. What is your nationality? (several choices possible)**

◻ France

◻ Switzerland

◻ Belgium

◻ Luxembourg

◻ Canada

◻ Other :

**1.2.3. Which country do you work in? (several choices possible)**

◻ France

◻ Switzerland

◻ Belgium

◻ Luxembourg

◻ Canada

◻ Other :

**1.2.4. What is your professional activity related to the use of the Hippocratic temperament theory? (several choices possible)**

◻ Naturopathic practitioner (patient/client therapy)

◻ Researcher

◻ Teacher

◻ Author

◻ Practitioner in another discipline (phytotherapy, dietetics, etc.)

◻ Other :

**1.2.5. What type of institution do you work for? (several choices possible)**

◻ Private practice

◻ School / educational institution ...

◻ University

◻ Laboratory

◻ Research center

◻ Other :

**1.2.6. How many years have you been practicing with the Hippocratic temperament theory?**

◻ Less than 5 years

◻ From 5 to 10 years

◻ More than 10 years

**1.3. LITERARY RESOURCES ON TEMPERAMENTS**

| **Literary resources on temperaments** | | | | | |
| --- | --- | --- | --- | --- | --- |
| 23 literary sources of traditional and contemporary texts on the subject of Hippocratic temperaments were used to prepare this questionnaire. They are listed below: | | | | | |
| **Buckingham, R. M.** (2002). Extraversion, neuroticism and the four temperaments of antiquity: an investigation of physiological reactivity. *Personality and Individual Differences, 2*(2), 225-246.  **Carton, P.** (1961). *Diagnostic et conduite des tempéraments*. Librairie Le François.  **Carton, P.** (1924). *Traité de médecine, d'alimentation et d'hygiène naturistes* (2^e^ éd.)*.* A. Maloine & Fils.  **Dammeyer, J. & Zettler, I.** (2018). A Brief Historical Overview on Links Between Personality and Health. In C. Johansen (Ed.), *Personality and Disease.* Academic Press. 1-16.  **Garvelmann, F.** (2018). *Konstitutionsmedizin*. BACOPA.  **Gex, M.** (1949). Les classifications des tempéraments. *Revue de Théologie et de Philosophie, 37(152),* 147-162.  **Gunsburger, N.** (2017). *Mon coach naturo : Mon programme sur mesure pour vivre en pleine santé.* Eyrolles.  **Howart, E.** (1988). Mood differences between the four Galen personality types: choleric, sanguine, phlegmatic, melancholic. *Personality and Individual Differences*, *9*(1)*, 173–175.* <https://doi.org/10.1016/0191-8869(88)90044-X>.  **Hutter, L.** (2017). *Diagnostic humoral de la langue*. Nhk Institut für integrative Naturheilkunde.  **Jouanna, J.** (2005). La théorie des quatre humeurs et des quatre tempéraments dans la tradition latine (Vindicien, Pseudo-Soranos) et une source grecque retrouvée. *Revue des Études Grecques*, *118*, 138-167.  **Kieffer, D.** (2004). *Guide personnel des bilans de santé : Encyclopédie naturopathique des tests morphologiques, psychologiques et biologiques de terrain (nouvelle éd.)*. Grancher.  **King, H. & Dasen, V.** (2008). *La médecine dans l’antiquité grecque et romaine*. Editions BHMS.  **Léaud-Zachoval, D.** (2017). *Quatre clefs pour la santé : Lymphatique, sanguin, bilieux, nerveux, qui suis-je vraiment ?* Editions Médicis  **Léaud-Zachoval, D.** (2021). Voyage au centre de la naturopathie. *Hippocrate*, *2*(5), 17-25.  **Osborn, D.** (2007). *Greek medicine*. Greekmedicine.net  **Openpsychometrics.** (2019). *OSPP Four Temperaments Test*. Openpsychometrics.org.  **Raimann, C., Ganz, C., Garvelmann, F., Bertischi-Stahl, H., & Fehr Streule, R.** (2017). *Grundlagen der Traditionellen Europäischen Naturheilkunde TEN.* BACOPA.  **Rolfe, R.** (2002). *The Four Temperaments*. Marlowe & Compagny.  **Ruch, W.** (1992). Pavlov’s Types of Nervous System, Eysenck’s Typology and the Hippocrates-Galen Temperaments: an Empirical Examination of the Asserted Correspondence of Three Temperament Typologies. In *Personality and individual Differences. 13*(12), 1259-1271. Pergamon Press Ldt.  **Salmani Nodoushan, M. A.** (2011). Temperament as an indicator of language achievement. *International Journal of Language Studies*. 5. 33-52.  **Ternisien, L.** (2020). *Naturopathie, le guide saison par saison*. Flammarion.  **Vanopdenbosch, Y.** (2012). *Les tempéraments : Outil de connaissance de soi et des autres*. Amyris.  **Voutsinas, D.** (1961). Tempérament, constitution, caractère. *Bulletin de psychologie*, *15*(197), 25-40. | | | | | |
| 1.3.1. If you wish to comment on one or more of these sources, please do so here, quoting the corresponding source number.  _______________________________________________________________________________ | | | | | |
| 1.3.2. Would you like to cite another source (article or book) that you feel is important or that you use in your naturopathic practice of the Hippocratic temperament theory?  _______________________________________________________________________________ | | | | | |

**1.4. CONCEPT OF TEMPERAMENT**

| **Concept of temperament** | | | | | |
| --- | --- | --- | --- | --- | --- |
| *Mark how strongly you agree with the following statements regarding the notion of Hippocratic temperament as applied in the current field of naturopathy. For the record, these statements are quotes from the literary resources.*  *1 Strongly Disagree, 2 Disagree, 3 Neutral, 4 Agree, 5 Strongly Agree* | | | | | |
|  | *1* | *2* | *3* | *4* | *5* |
| 1.4.1. In practice, we describe four main temperaments: Lymphatic (or phlegmatic or pituitary), sanguine, choleric and melancholic. | ◻ | ◻ | ◻ | ◻ | ◻ |
| 1.4.2. The four temperaments are our uniquely human way of expressing the balance of four forces that govern all thing according to the ancient way of understanding character and health. These four forces are the humors, which mix and move within us and affect every aspect of our lives. | ◻ | ◻ | ◻ | ◻ | ◻ |
| 1.4.3. A person's temperament is the combination of the humors that compose them. | ◻ | ◻ | ◻ | ◻ | ◻ |
| 1.4.4. The temperament is one of the expressions of the terrain. | ◻ | ◻ | ◻ | ◻ | ◻ |
| 1.4.5. The temperament is the activity of vital forces as it manifests in each individual. | ◻ | ◻ | ◻ | ◻ | ◻ |
| 1.4.6. Actual individuals most often belong to several types, sometimes with a marked dominance. It can be said that an individual is never a pure type. | ◻ | ◻ | ◻ | ◻ | ◻ |
| 1.4.7. We are always imbued with a fundamental temperament that characterizes us, but it can fade in favor of one or more temperaments that emerge over the course of our life. | ◻ | ◻ | ◻ | ◻ | ◻ |
| 1.4.8. Temperament is that aspect of our personalities that is genetically based, inborn, there from birth or even before. | ◻ | ◻ | ◻ | ◻ | ◻ |
| 1.4.9. Temperament is inherently innate, but it also stems from the acquired. | ◻ | ◻ | ◻ | ◻ | ◻ |
| 1.4.10. Temperament is far from being fixed, it is a reflection of life, it is an ongoing dynamic that is sometimes excessively or insufficiently expressed. | ◻ | ◻ | ◻ | ◻ | ◻ |
| 1.4.11. Temperament in itself has not pathological significance. However, the dominating humor tends to be a potential pathogen if stimuli can no longer be compensated. | ◻ | ◻ | ◻ | ◻ | ◻ |
| 1.4.12. If they [temperaments] are on an uncertain path, their relative dynamics show more of a mixture of the four constitutional values distributed individually, than a true evolutionary or involutionary shift from one temperament to another. | ◻ | ◻ | ◻ | ◻ | ◻ |
| 1.4.13. Temperament is not definitive, it evolves over the course of life in accordance with lived situations. | ◻ | ◻ | ◻ | ◻ | ◻ |
| 1.4.14. Temperament is the main setting of the constitution. | ◻ | ◻ | ◻ | ◻ | ◻ |
| 1.4.15. The four temperaments are the basic constitutional bodymind types of Greek Medicine. | ◻ | ◻ | ◻ | ◻ | ◻ |
| 1.4.16. Extraversion (E, the tendency to enjoy social events and interaction) and Neuroticism (N, the tendency to experience negative emotions) [...] reflect Hippocrates’ four temperaments. | ◻ | ◻ | ◻ | ◻ | ◻ |
| 1.4.17. Temperament expresses individual unity that encompasses physiological, morphological and psychological traits. | ◻ | ◻ | ◻ | ◻ | ◻ |
| 1.4.18. The four fundamental tendencies or temperaments (sanguine, phlegmatic, choleric, melancholic) correspond to the development of the four primordial instincts (breathing, eating, moving, thinking) which trigger a preference in the search for certain vital stimulants (air, food, exercise, psychic excitement) according to individual predominances. | ◻ | ◻ | ◻ | ◻ | ◻ |

**1.5. TEMPERAMENT QUADRANT**

| **Temperament Quadrant** | | | | | |
| --- | --- | --- | --- | --- | --- |
| *After observing this quadrant representation, indicate to what extent you agree with the following statements concerning the notion of Hippocratic temperament and its associated qualities.* | | | | | |
| **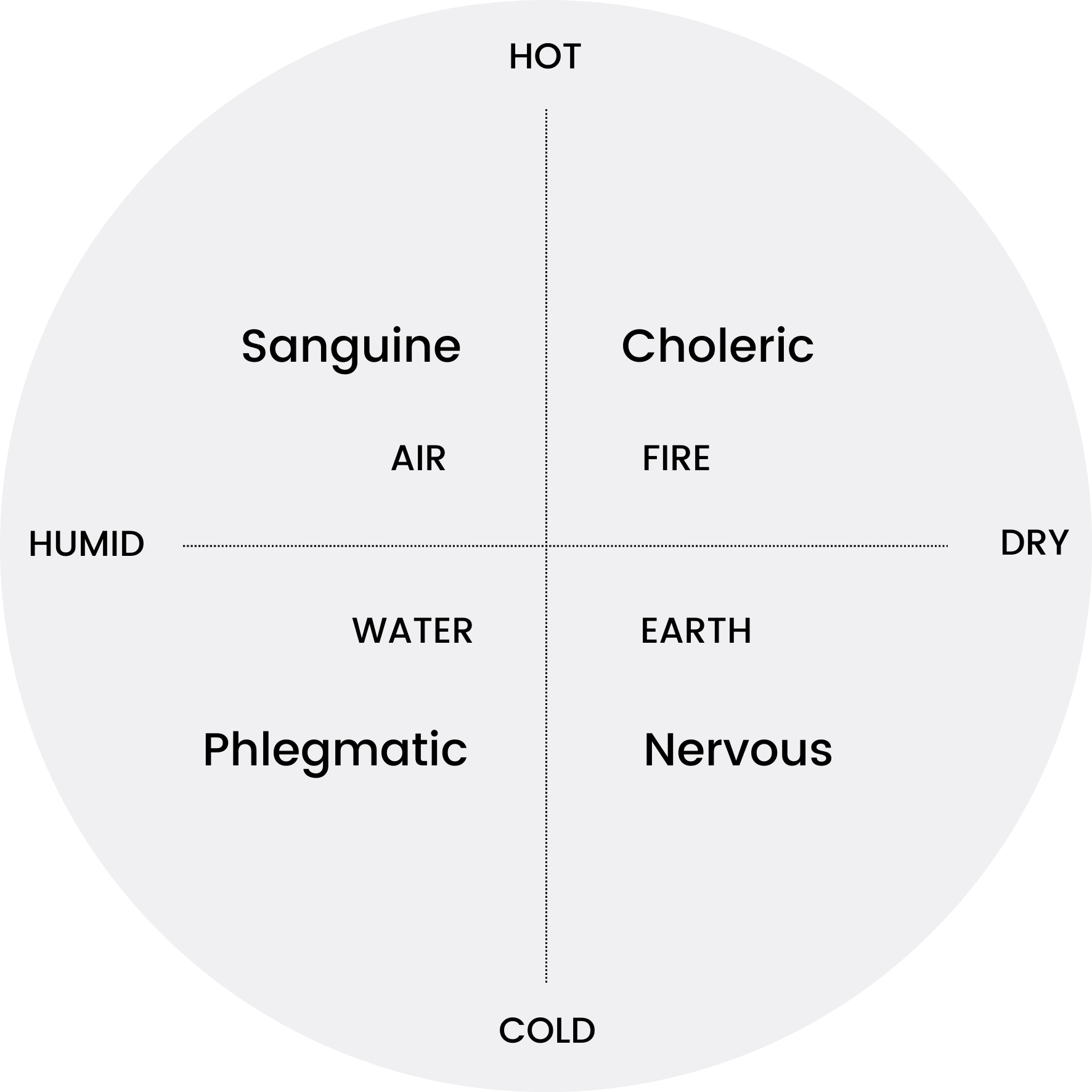** | | | | | |
| *1 Strongly Disagree, 2 Disagree, 3 Neutral, 4 Agree, 5 Strongly Agree* | *1* | *2* | *3* | *4* | *5* |
| 1.5.1. In the presented diagram, do you agree with the distribution of the elements (earth/water/fire/air) associated with each temperament? | ◻ | ◻ | ◻ | ◻ | ◻ |
| 1.5.2. In the presented diagram, do you agree with the distribution of the qualities (hot/dry/cold/moist) associated with each temperament? | ◻ | ◻ | ◻ | ◻ | ◻ |
| 1.5.3. Do you agree with the fact that the moisture principle is associated with dilation (governs phlegmatic and sanguine profiles)? | ◻ | ◻ | ◻ | ◻ | ◻ |
| 1.5.4. Do you agree with the fact that the dryness principle is associated with constriction (governs choleric and melancholic profiles)? | ◻ | ◻ | ◻ | ◻ | ◻ |

**1.6. DIMENSIONS OF TEMPERAMENT ASSESSMENT**

| **Dimensions of temperament assessment** | | | | | |
| --- | --- | --- | --- | --- | --- |
| *Of the various temperament assessment dimensions listed below, which do you feel are the most important to consider in your current naturopathic practice? Please rank each dimension from 1 to 5 (1=less important to 5=very important). Multiple dimensions may be rated with the same importance (example: 4-4-3-2-5).* | | | | | |
|  | *1* | *2* | *3* | *4* | *5* |
| 1.6.1. Psycho-emotional dimension  Psychic and emotional experiences | ◻ | ◻ | ◻ | ◻ | ◻ |
| 1.6.2. Behavioral dimension  Behaviors and reactions | ◻ | ◻ | ◻ | ◻ | ◻ |
| 1.6.3. Physiological dimension  Physiological functioning of the body in the absence of disease (sleep, appetite, weight gain, coldness, sweating...) | ◻ | ◻ | ◻ | ◻ | ◻ |
| 1.6.4. Morphological dimension  Physical appearance (general, body, face, hand, tongue...) | ◻ | ◻ | ◻ | ◻ | ◻ |
| 1.6.5. Susceptibility to diseases  Vulnerability to specific and recurrent pathologies | ◻ | ◻ | ◻ | ◻ | ◻ |
| 1.6.6. What other dimension of evaluating a Hippocratic temperament do you think is important? **________________________________________________________________________________** | | | | | |

The questionnaire is now complete. You can still go back and edit your answers if you wish to do so. If you have finished, you must click on the next page to validate your participation in this round of the questionnaire.

Thank you very much for taking part in this study!

Take care.

The Navi and UTS team.

SECOND ROUND OF DELPHI QUESTIONNAIRE (2/4)

**2.1. INTRODUCTION**

## **Presentation of first round results**

We thank you for your participation in the first round and are pleased to present some results that are important for the second round. Please read the information below before proceeding with the rest of the survey.

1. Concerning the temperament concept, some proposals from the previous round were rejected on the basis of at least 50% disagreement. Others were validated on the basis of at least 75% agreement, and the remaining elements will be proposed again in this second round.

The elements accepted as validated by your community of experts at this stage are as follows:

- In practice, we describe four main temperaments: Lymphatic (or phlegmatic or pituitary), sanguine, choleric and melancholic.
- The four temperaments are our uniquely human way of expressing the balance of four forces that govern all thing according to the ancient way of understanding character and health. These four forces are the humors, which mix and move within us and affect every aspect of our lives.
- A person's temperament is the combination of the humors that compose them.
- The temperament is one of the expressions of the terrain.
- Actual individuals most often belong to several types, sometimes with a marked dominance. It can be said that an individual is never a pure type.
- We are always imbued with a fundamental temperament that characterizes us, but it can fade in favor of one or more temperaments that emerge over the course of our life.
- Temperament is far from being fixed, it is a reflection of life, it is an ongoing dynamic that is sometimes excessively or insufficiently expressed.
- Temperament in itself has not pathological significance. However, the dominating humor tends to be a potential pathogen if stimuli can no longer be compensated.
- Temperament is not definitive, it evolves over the course of life in accordance with lived situations.
- Temperament expresses individual unity that encompasses physiological, morphological and psychological traits.

2. **The temperament quadrant is validated with over 75% agreement by your community as follows:**


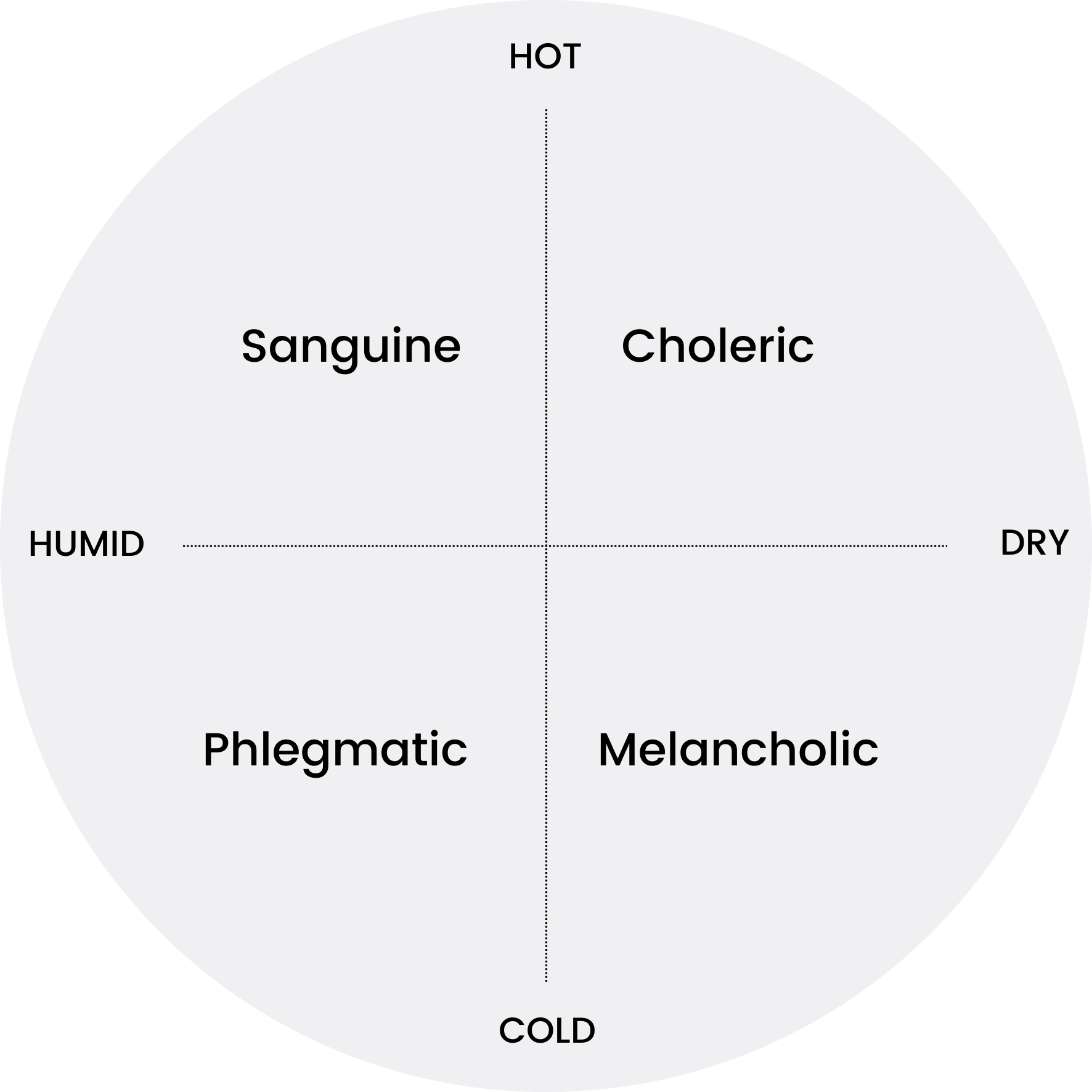


The association of the four elements with each temperament according to the Water-Phlegmatic, Air-Sanguine, Fire-Choleric and Earth-Melancholic model, has not been consensually accepted at this stage.

3. The average importance of the different temperament dimensions is evaluated as follows:


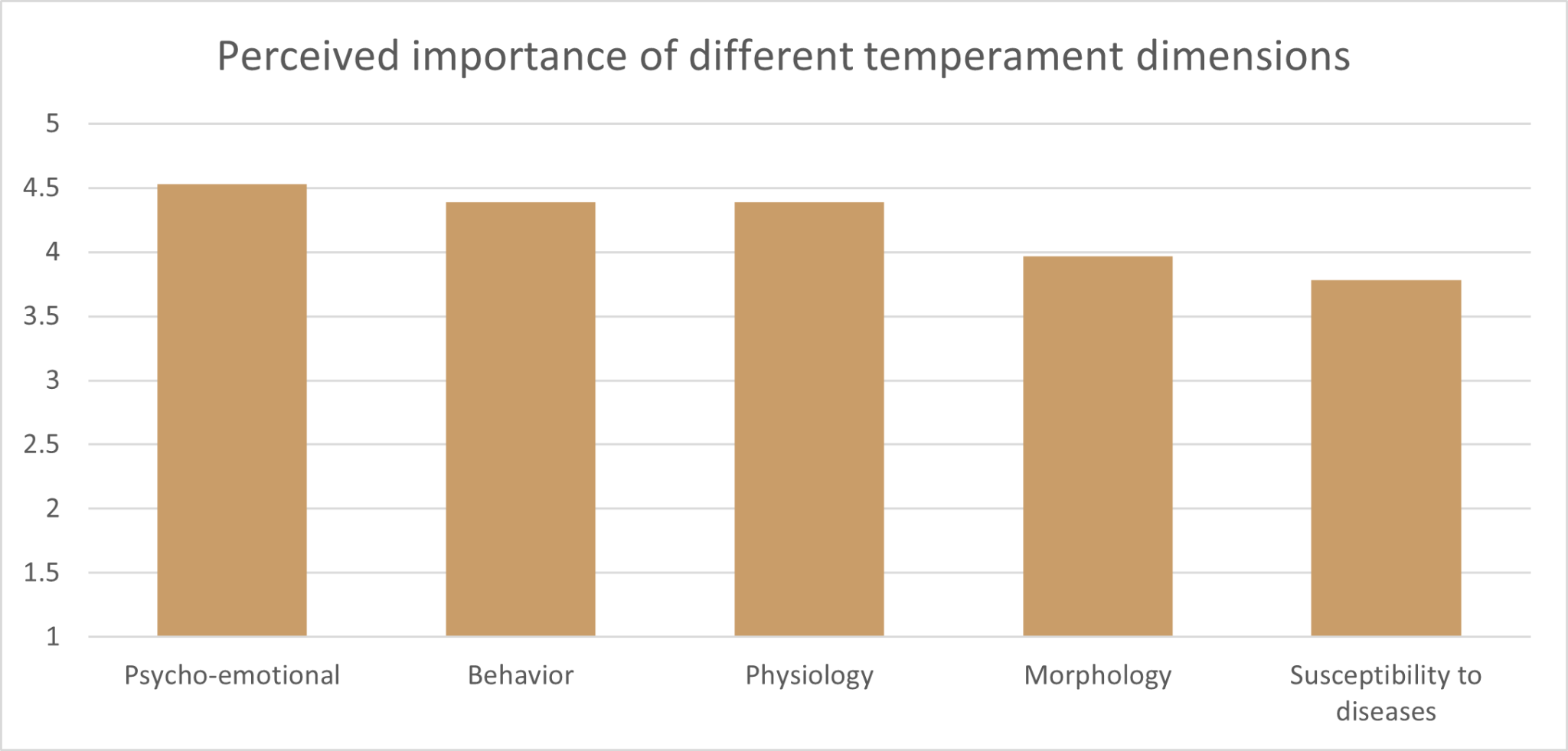


## **Objectives and content of the second questionnaire**

The aim of the next questionnaire you will complete is still to find a consensus that reflects the reality of the practice and teaching of this theory in the French-speaking naturopathic community.

1. For this new round, we need to collect your general information again.

2. In a second step, some of the proposals from the first round will be presented to you again, so that you can evaluate them in the light of the results of the first round.

3. Third, you will be given statements to assess or characterize the dominance of the lymphatic, sanguine, choleric and melancholic temperaments.

These suggestions are quotations and reformulations based on data in the literature. We ask you to specify the extent to which they reflect your theoretical and/or practical experience. Further proposals will be presented in rounds 3 and 4 of the study.

You always have the opportunity to add your thoughts and comments in the “Comments” section. Please note that after each round, we take your answers and comments into account to build the questionnaire for the next round and thus refine the consensus.

**Participant information sheet**

By using this link, you can return to the participant information sheet sent to you during recruitment. We remind you that submitting the questionnaire online is an indication of your consent.

## **2.2. ABOUT YOU**

Before we start, we need to gather some information about your practice again, to ensure that we collect a wide variety of perspectives on the subject, as well as to be able to relate these perspectives to the study results.

## **2.2.1. Which genre do you identify with?**

◻ Female

◻ Male

◻ Other

**2.2.2. What is your nationality? (several choices possible)**

◻ France

◻ Switzerland

◻ Belgium

◻ Luxembourg

◻ Canada

◻ Other :

**2.2.3. Which country do you work in? (several choices possible)**

◻ France

◻ Switzerland

◻ Belgium

◻ Luxembourg

◻ Canada

◻ Other :

**2.2.4. What is your professional activity related to the use of the Hippocratic temperament theory? (several choices possible)**

◻ Naturopathic practitioner (patient/client therapy)

◻ Researcher

◻ Teacher

◻ Author

◻ Practitioner in another discipline (phytotherapy, dietetics, etc.)

◻ Other :

**2.2.5. What type of institution do you work for? (several choices possible)**

◻ Private practice

◻ School / educational institution ...

◻ University

◻ Laboratory

◻ Research center

◻ Other :

**2.2.6. How many years have you been practicing with the Hippocratic temperament theory?**

◻ Less than 5 years

◻ From 5 to 10 years

◻ More than 10 years

**2.3. LITERARY RESOURCES ON TEMPERAMENTS**

| **Literary resources on temperaments** | | | | | |
| --- | --- | --- | --- | --- | --- |
| 24 literary sources of traditional and contemporary texts on the subject of Hippocratic temperaments were used to prepare this questionnaire. They are listed below: | | | | | |
| **Buckingham, R. M.** (2002). Extraversion, neuroticism and the four temperaments of antiquity: an investigation of physiological reactivity. *Personality and Individual Differences, 2*(2), 225-246.  **Carton, P.** (1961). *Diagnostic et conduite des tempéraments*. Librairie Le François.  **Carton, P.** (1984). *Traité de médecine, d'alimentation et d'hygiène naturistes* (4^e^ éd.)*.* A. Maloine & Fils.  **Dammeyer, J. & Zettler, I.** (2018). A Brief Historical Overview on Links Between Personality and Health. In C. Johansen (Ed.), *Personality and Disease.* Academic Press. 1-16.  **Garvelmann, F.** (2018). *Konstitutionsmedizin*. BACOPA.  **Gex, M.** (1949). Les classifications des tempéraments. *Revue de Théologie et de Philosophie, 37(152),* 147-162.  **Gunsburger, N.** (2017). *Mon coach naturo : Mon programme sur mesure pour vivre en pleine santé.* Eyrolles.  **Howart, E.** (1988). Mood differences between the four Galen personality types: choleric, sanguine, phlegmatic, melancholic. *Personality and Individual Differences*, *9*(1)*, 173–175.* <https://doi.org/10.1016/0191-8869(88)90044-X>.  **Hutter, L.** (2017). *Diagnostic humoral de la langue*. Nhk Institut für integrative Naturheilkunde.  **Jouanna, J.** (2005). La théorie des quatre humeurs et des quatre tempéraments dans la tradition latine (Vindicien, Pseudo-Soranos) et une source grecque retrouvée. *Revue des Études Grecques*, *118*, 138-167.  **Kieffer, D.** (2004). *Guide personnel des bilans de santé : Encyclopédie naturopathique des tests morphologiques, psychologiques et biologiques de terrain (nouvelle éd.)*. Grancher.  **King, H. & Dasen, V.** (2008). *La médecine dans l’antiquité grecque et romaine*. Editions BHMS.  **Léaud-Zachoval, D.** (2017). *Quatre clefs pour la santé : Lymphatique, sanguin, bilieux, nerveux, qui suis-je vraiment ?* Editions Médicis  **Léaud-Zachoval, D.** (2021). Voyage au centre de la naturopathie. *Hippocrate*, *2*(5), 17-25.  **Osborn, D.** (2007). *Greek medicine*. Greekmedicine.net  **Openpsychometrics.** (2019). *OSPP Four Temperaments Test*. Openpsychometrics.org.  **Raimann, C., Ganz, C., Garvelmann, F., Bertischi-Stahl, H., & Fehr Streule, R.** (2017). *Grundlagen der Traditionellen Europäischen Naturheilkunde TEN.* BACOPA.  **Rolfe, R.** (2002). *The Four Temperaments*. Marlowe & Compagny.  **Ruch, W.** (1992). Pavlov’s Types of Nervous System, Eysenck’s Typology and the Hippocrates-Galen Temperaments: an Empirical Examination of the Asserted Correspondence of Three Temperament Typologies. In *Personality and individual Differences. 13*(12), 1259-1271. Pergamon Press Ldt.  **Salmani Nodoushan, M. A.** (2011). Temperament as an indicator of language achievement. *International Journal of Language Studies*. 5. 33-52.  **Steiner, R, Laloux, M., Berthold-Andrea, H., Schad, W. & Smit, J.** (2014). L'énigme des tempéraments. Triades.  **Ternisien, L.** (2020). *Naturopathie, le guide saison par saison*. Flammarion.  **Vanopdenbosch, Y.** (2012). *Les tempéraments : Outil de connaissance de soi et des autres*. Amyris.  **Voutsinas, D.** (1961). Tempérament, constitution, caractère. *Bulletin de psychologie*, *15*(197), 25-40. | | | | | |

**2.4. CONCEPT OF TEMPERAMENT**

| **Concept of temperament** | | | | | |
| --- | --- | --- | --- | --- | --- |
| *Please indicate again to what extent you agree with the following statements concerning the notion of Hippocratic temperament as applied in the current field of naturopathy.**For the record, these statements are quotations from the literary resources presented above.* *1 Strongly Disagree, 2 Disagree, 3 Neutral, 4 Agree, 5 Strongly Agree* | | | | | |
|  | *1* | *2* | *3* | *4* | *5* |
| 2.4.1. The temperament is the activity of vital forces as it manifests in each individual. | ◻ | ◻ | ◻ | ◻ | ◻ |
| 2.4.2. Temperament is inherently innate, but it also stems from the acquired. | ◻ | ◻ | ◻ | ◻ | ◻ |
| 2.4.3. The four temperaments are the basic constitutional bodymind types of Greek Medicine. | ◻ | ◻ | ◻ | ◻ | ◻ |
| 2.4.4. The four fundamental tendencies or temperaments (sanguine, phlegmatic, choleric, melancholic) correspond to the development of the four primordial instincts (breathing, eating, moving, thinking) which trigger a preference in the search for certain vital stimulants (air, food, exercise, psychic excitement) according to individual predominances. | ◻ | ◻ | ◻ | ◻ | ◻ |

**2.5. PSYCHO-EMOTIONAL DIMENSION**

| **Psycho-emotional dimension** | | | | | |
| --- | --- | --- | --- | --- | --- |
| *Mark how strongly you agree with the following statements regarding a person's psychological and emotional tendencies according to their dominant temperament.*  *1 Strongly Disagree, 2 Disagree, 3 Neutral, 4 Agree, 5 Strongly Agree.* | | | | | |
| *A person of* ***phlegmatic*** *dominance will tend to:* | | | | | |
|  | *1* | *2* | *3* | *4* | *5* |
| 2.5.1. Appreciate the calm | ◻ | ◻ | ◻ | ◻ | ◻ |
| 2.5.2. Be emotionally stable by nature | ◻ | ◻ | ◻ | ◻ | ◻ |
| 2.5.3. Appreciate slowness and take his/her time | ◻ | ◻ | ◻ | ◻ | ◻ |
| 2.5.4. Be introverted by nature | ◻ | ◻ | ◻ | ◻ | ◻ |
| 2.5.5. Have an intuitive intelligence | ◻ | ◻ | ◻ | ◻ | ◻ |
| 2.5.6. Be imaginative | ◻ | ◻ | ◻ | ◻ | ◻ |
| Comments: ___________________________________________________________________ | | | | | |
| *A person of* ***sanguine*** *dominance will tend to:* | | | | | |
|  | *1* | *2* | *3* | *4* | *5* |
| 2.5.7. Be optimistic | ◻ | ◻ | ◻ | ◻ | ◻ |
| 2.5.8. Be extroverted by nature | ◻ | ◻ | ◻ | ◻ | ◻ |
| 2.5.9. Be curious by nature | ◻ | ◻ | ◻ | ◻ | ◻ |
| 2.5.10. Be emotionally explosive by nature | ◻ | ◻ | ◻ | ◻ | ◻ |
| 2.5.11. Be sensual and passionate by nature | ◻ | ◻ | ◻ | ◻ | ◻ |
| 2.5.12. Have an epicurean spirit |  |  |  |  |  |
| Comments: ___________________________________________________________________ | | | | | |
| *A person of* ***choleric*** *dominance will tend to:* | | | | | |
|  | *1* | *2* | *3* | *4* | *5* |
| 2.5.13. Be energetic by nature | ◻ | ◻ | ◻ | ◻ | ◻ |
| 2.5.14. Be extroverted by nature | ◻ | ◻ | ◻ | ◻ | ◻ |
| 2.5.15. Present changing emotions | ◻ | ◻ | ◻ | ◻ | ◻ |
| 2.5.16. Be determined by nature | ◻ | ◻ | ◻ | ◻ | ◻ |
| 2.5.17. Enjoy debating | ◻ | ◻ | ◻ | ◻ | ◻ |
| Comments: ___________________________________________________________________ | | | | | |
| *A person of* ***melancholic*** *dominance will tend to:* | | | | | |
|  | *1* | *2* | *3* | *4* | *5* |
| 2.5.18. Be introverted by nature | ◻ | ◻ | ◻ | ◻ | ◻ |
| 2.5.19. Feel fluctuations in emotions | ◻ | ◻ | ◻ | ◻ | ◻ |
| 2.5.20. Have a sharp intelligence and a good analytical mind | ◻ | ◻ | ◻ | ◻ | ◻ |
| 2.5.21. Appreciate detail and precision | ◻ | ◻ | ◻ | ◻ | ◻ |
| 2.5.22. Be pessimistic by nature | ◻ | ◻ | ◻ | ◻ | ◻ |
| Comments: ___________________________________________________________________ | | | | | |

**2.6. BEHAVIORAL DIMENSION**

| **Behavioral dimension** | | | | | |
| --- | --- | --- | --- | --- | --- |
| *Mark how strongly you agree with the following statements regarding a person's behavioral tendencies according to their dominant temperament.*  *1 Strongly Disagree, 2 Disagree, 3 Neutral, 4 Agree, 5 Strongly Agree* | | | | | |
| *A person of* ***phlegmatic*** *dominance will tend to:* | | | | | |
|  | *1* | *2* | *3* | *4* | *5* |
| 2.6.1. Take his/her/they time before reacting to external stimuli | ◻ | ◻ | ◻ | ◻ | ◻ |
| 2.6.2. Be affectionate | ◻ | ◻ | ◻ | ◻ | ◻ |
| 2.6.3. Be conciliatory | ◻ | ◻ | ◻ | ◻ | ◻ |
| 2.6.4. Have a great capacity of adaptation | ◻ | ◻ | ◻ | ◻ | ◻ |
| 2.6.5. Prefer stability to change and predictability to uncertainty | ◻ | ◻ | ◻ | ◻ | ◻ |
| Comments: ___________________________________________________________________ | | | | | |
| *A person of* ***sanguine*** *dominance will tend to:* | | | | | |
|  | *1* | *2* | *3* | *4* | *5* |
| 2.6.6. Be sociable | ◻ | ◻ | ◻ | ◻ | ◻ |
| 2.6.7. Be communicative | ◻ | ◻ | ◻ | ◻ | ◻ |
| 2.6.8. Be motivated by pleasure | ◻ | ◻ | ◻ | ◻ | ◻ |
| 2.6.9. Enjoy making others laugh | ◻ | ◻ | ◻ | ◻ | ◻ |
| 2.6.10. Make decisions by being governed by affect | ◻ | ◻ | ◻ | ◻ | ◻ |
| Comments: ___________________________________________________________________ | | | | | |
| *A person of* ***choleric*** *dominance will tend to:* | | | | | |
|  | *1* | *2* | *3* | *4* | *5* |
| 2.6.11. Being active, even hyperactive | ◻ | ◻ | ◻ | ◻ | ◻ |
| 2.6.12. Be comfortable in a leadership role | ◻ | ◻ | ◻ | ◻ | ◻ |
| 2.6.13. Be organized | ◻ | ◻ | ◻ | ◻ | ◻ |
| 2.6.14. Have a need for control | ◻ | ◻ | ◻ | ◻ | ◻ |
| 2.6.15. Be persistent | ◻ | ◻ | ◻ | ◻ | ◻ |
| Comments: ___________________________________________________________________ | | | | | |
| *A person of* ***melancholic*** *dominance will tend to:* | | | | | |
|  | *1* | *2* | *3* | *4* | *5* |
| 2.6.16. Enjoy intellectual or artistic professions | ◻ | ◻ | ◻ | ◻ | ◻ |
| 2.6.17. Seek perfection | ◻ | ◻ | ◻ | ◻ | ◻ |
| 2.6.18. Eat small amounts | ◻ | ◻ | ◻ | ◻ | ◻ |
| 2.6.19. Be punctual | ◻ | ◻ | ◻ | ◻ | ◻ |
| 2.6.20. Organize, tidy up and structure his/her/they environment | ◻ | ◻ | ◻ | ◻ | ◻ |
| Comments: ___________________________________________________________________ | | | | | |

**2.7. PHYSIOLOGICAL DIMENSION**

| **Physiological dimension** | | | | | |
| --- | --- | --- | --- | --- | --- |
| *Mark how strongly you agree with the following statements regarding a person's physiological tendencies (in the sense of normal body functions and reactions) according to their dominant temperament.*  *1 Strongly Disagree, 2 Disagree, 3 Neutral, 4 Agree, 5 Strongly Agree.* | | | | | |
| *A person of* ***phlegmatic*** *dominance will tend to:* | | | | | |
|  | *1* | *2* | *3* | *4* | *5* |
| 2.7.1. Lack of energy | ◻ | ◻ | ◻ | ◻ | ◻ |
| 2.7.2. Need many hours of sleep and sometimes sleeping excessively | ◻ | ◻ | ◻ | ◻ | ◻ |
| 2.7.3. Have a slow digestion | ◻ | ◻ | ◻ | ◻ | ◻ |
| 2.7.4. Have an anabolic metabolism, which tends to store or accumulate, especially fat reserves | ◻ | ◻ | ◻ | ◻ | ◻ |
| 2.7.5. Have a rather strong appetite | ◻ | ◻ | ◻ | ◻ | ◻ |
| Comments: ___________________________________________________________________ | | | | | |
| *A person of* ***sanguine*** *dominance will tend to:* | | | | | |
|  | *1* | *2* | *3* | *4* | *5* |
| 2.7.6. Have a good vitality | ◻ | ◻ | ◻ | ◻ | ◻ |
| 2.7.7. Be warm | ◻ | ◻ | ◻ | ◻ | ◻ |
| 2.7.8. Have a good, deep and balanced sleep | ◻ | ◻ | ◻ | ◻ | ◻ |
| 2.7.9. Have good digestive capacities | ◻ | ◻ | ◻ | ◻ | ◻ |
| 2.7.10. Sweat profusely | ◻ | ◻ | ◻ | ◻ | ◻ |
| Comments: ___________________________________________________________________ | | | | | |
| *A person of* ***choleric*** *dominance will tend to:* | | | | | |
|  | *1* | *2* | *3* | *4* | *5* |
| 2.7.11. Have a big appetite | ◻ | ◻ | ◻ | ◻ | ◻ |
| 2.7.12. Have a hot metabolism | ◻ | ◻ | ◻ | ◻ | ◻ |
| 2.7.13. Have a recuperative sleep | ◻ | ◻ | ◻ | ◻ | ◻ |
| 2.7.14. Have a high vital force | ◻ | ◻ | ◻ | ◻ | ◻ |
| 2.7.15. Accumulate little fat | ◻ | ◻ | ◻ | ◻ | ◻ |
| Comments: ___________________________________________________________________ | | | | | |
| *A person of melancholic dominance will tend to:* | | | | | |
|  | *1* | *2* | *3* | *4* | *5* |
| 2.7.16. Be chilly | ◻ | ◻ | ◻ | ◻ | ◻ |
| 2.7.17. Eat little and often | ◻ | ◻ | ◻ | ◻ | ◻ |
| 2.7.18. Lack energy | ◻ | ◻ | ◻ | ◻ | ◻ |
| 2.7.19. Lose weight easily | ◻ | ◻ | ◻ | ◻ | ◻ |
| 2.7.20. Sweating little | ◻ | ◻ | ◻ | ◻ | ◻ |
| Comments: ___________________________________________________________________ | | | | | |

**2.8. MORPHOLOGICAL DIMENSION**

| **Morphological dimension** | | | | | |
| --- | --- | --- | --- | --- | --- |
| *Mark how strongly you agree with the following statements regarding a person's physical traits according to their dominant temperament.*  *1 Strongly Disagree, 2 Disagree, 3 Neutral, 4 Agree, 5 Strongly Agree.* | | | | | |
| *A person of* ***phlegmatic*** *dominance will tend to:* | | | | | |
|  | *1* | *2* | *3* | *4* | *5* |
| 2.8.1. Be of brevilinear build (i.e. with a rather long trunk and rather short limbs) | ◻ | ◻ | ◻ | ◻ | ◻ |
| 2.8.2. Have rather broad forms | ◻ | ◻ | ◻ | ◻ | ◻ |
| 2.8.3. Have a fresh and wet skin | ◻ | ◻ | ◻ | ◻ | ◻ |
| 2.8.4. To have a rounded face | ◻ | ◻ | ◻ | ◻ | ◻ |
| 2.8.5. Have a pale complexion | ◻ | ◻ | ◻ | ◻ | ◻ |
| 2.8.6. Have a moist tongue that may be swollen or edematous | ◻ | ◻ | ◻ | ◻ | ◻ |
| 2.8.7. Have a large pale tongue | ◻ | ◻ | ◻ | ◻ | ◻ |
| 2.8.8. Have square fingers | ◻ | ◻ | ◻ | ◻ | ◻ |
| 2.8.9. Have short, chubby hands | ◻ | ◻ | ◻ | ◻ | ◻ |
| Comments: ___________________________________________________________________ | | | | | |
| *A person of* ***sanguine*** *dominance will tend to:* | | | | | |
|  | *1* | *2* | *3* | *4* | *5* |
| 2.8.10. Have a brevilinear build (i.e. with a rather long trunk and rather short limbs) | ◻ | ◻ | ◻ | ◻ | ◻ |
| 2.8.11. Be muscular | ◻ | ◻ | ◻ | ◻ | ◻ |
| 2.8.12. Have a warm and wet skin | ◻ | ◻ | ◻ | ◻ | ◻ |
| 2.8.13. Have a more developed mid-face (the part between the top of the eyebrows and the line marking the lower edge of the nose and cheekbones) | ◻ | ◻ | ◻ | ◻ | ◻ |
| 2.8.14. Have a reddish complexion | ◻ | ◻ | ◻ | ◻ | ◻ |
| 2.8.15. Have a large, sometimes swollen tongue | ◻ | ◻ | ◻ | ◻ | ◻ |
| 2.8.16. Have a pink to red tongue, sometimes even purple | ◻ | ◻ | ◻ | ◻ | ◻ |
| 2.8.17. Have large, fleshy hands | ◻ | ◻ | ◻ | ◻ | ◻ |
| 2.8.18. To have short and strong fingers | ◻ | ◻ | ◻ | ◻ | ◻ |
| Comments: ___________________________________________________________________ | | | | | |
| *A person of* ***choleric*** *dominance will tend to:* | | | | | |
|  | *1* | *2* | *3* | *4* | *5* |
| 2.8.19. Have a long body shape (i.e. with a rather short trunk and rather long limbs) | ◻ | ◻ | ◻ | ◻ | ◻ |
| 2.8.20. Have a toned, athletic and muscular body | ◻ | ◻ | ◻ | ◻ | ◻ |
| 2.8.21. Have a warm and dry skin | ◻ | ◻ | ◻ | ◻ | ◻ |
| 2.8.22. Have a square or rectangular face | ◻ | ◻ | ◻ | ◻ | ◻ |
| 2.8.23. Have a dark or tanned skin | ◻ | ◻ | ◻ | ◻ | ◻ |
| 2.8.24. Have a red tongue or have red spots on the surface | ◻ | ◻ | ◻ | ◻ | ◻ |
| 2.8.25. Have a dry tongue with grooves or cracks | ◻ | ◻ | ◻ | ◻ | ◻ |
| 2.8.26. Have a square or rectangular hand shape | ◻ | ◻ | ◻ | ◻ | ◻ |
| 2.8.27. Having fingers that are equal to or longer than the palm of the hands | ◻ | ◻ | ◻ | ◻ | ◻ |
| Comments: ___________________________________________________________________ | | | | | |
| *A person of* ***melancholic*** *dominance will tend to:* | | | | | |
|  | *1* | *2* | *3* | *4* | *5* |
| 2.8.28. Be thin | ◻ | ◻ | ◻ | ◻ | ◻ |
| 2.8.29. Be of long body shape (i.e. with a rather short trunk and rather long limbs) | ◻ | ◻ | ◻ | ◻ | ◻ |
| 2.8.30. Have a pale skin | ◻ | ◻ | ◻ | ◻ | ◻ |
| 2.8.31. Have a shifty and worried | ◻ | ◻ | ◻ | ◻ | ◻ |
| 2.8.32. Have the upper level of the face (the part included between the eyebrows and the base of the hair) more dominant by its dilation, its height and/or its width | ◻ | ◻ | ◻ | ◻ | ◻ |
| 2.8.33. Present a small, firm and pointed tongue | ◻ | ◻ | ◻ | ◻ | ◻ |
| 2.8.34. Present a pale or bluish colored tongue | ◻ | ◻ | ◻ | ◻ | ◻ |
| 2.8.35. Show hands that become progressively thinner to the fingertips | ◻ | ◻ | ◻ | ◻ | ◻ |
| 2.8.36. Show long, thin, gnarled fingers that may be hyperlaxed | ◻ | ◻ | ◻ | ◻ | ◻ |
| Comments: ___________________________________________________________________ | | | | | |

**2.9. SUSCEPTIBILITY TO DISEASES**

| **Susceptibility to diseases** | | | | | |
| --- | --- | --- | --- | --- | --- |
| *Mark how strongly you agree with the following statements regarding a person's vulnerability to disease and imbalance, according to their dominant temperament.*  *1 Strongly Disagree, 2 Disagree, 3 Neutral, 4 Agree, 5 Strongly Agree.* | | | | | |
| *A person of* ***phlegmatic*** *dominance in imbalance will tend to:* | | | | | |
|  | 1 | 2 | 3 | 4 | 5 |
| 2.9.1. Express hypofunctional pathologies | ◻ | ◻ | ◻ | ◻ | ◻ |
| 2.9.2. Present fragility of the lymphatic system such as oedema or water retention | ◻ | ◻ | ◻ | ◻ | ◻ |
| 2.9.3. Present skin disorders | ◻ | ◻ | ◻ | ◻ | ◻ |
| 2.9.4. Express ENT pathologies | ◻ | ◻ | ◻ | ◻ | ◻ |
| 2.9.5. Be prone to diabetes | ◻ | ◻ | ◻ | ◻ | ◻ |
| Comments: ___________________________________________________________________ | | | | | |
| *A person of* ***sanguine*** *dominance in imbalance will tend to:* | | | | | |
|  | 1 | 2 | 3 | 4 | 5 |
| 2.9.6. Have cardiovascular fragility, such as hypertension, tachycardia, hot flashes, atherosclerosis, congestive migraine or infarction | ◻ | ◻ | ◻ | ◻ | ◻ |
| 2.9.7. Present fragility in the respiratory sphere, such as pneumonia, asthma, fever | ◻ | ◻ | ◻ | ◻ | ◻ |
| 2.9.8. Be prone to circulatory problems, such as varicose veins, phlebitis or hemorrhoids | ◻ | ◻ | ◻ | ◻ | ◻ |
| 2.9.9. Develop metabolic pathologies, such as diabetes, gout, uremia or hypercholesterolemia | ◻ | ◻ | ◻ | ◻ | ◻ |
| 2.9.10. Becoming acutely ill | ◻ | ◻ | ◻ | ◻ | ◻ |
| Comments: ___________________________________________________________________ | | | | | |
| *A person of* ***choleric*** *dominance in imbalance will tend to:* | | | | | |
|  | 1 | 2 | 3 | 4 | 5 |
| 2.9.11. Have liver and gallbladder imbalances | ◻ | ◻ | ◻ | ◻ | ◻ |
| 2.9.12. Suffer from insomnia with incessant thoughts | ◻ | ◻ | ◻ | ◻ | ◻ |
| 2.9.13. Have arthritis | ◻ | ◻ | ◻ | ◻ | ◻ |
| 2.9.14. Express nervous digestive disorders | ◻ | ◻ | ◻ | ◻ | ◻ |
| 2.9.15. Be prone to nutritional deficiencies | ◻ | ◻ | ◻ | ◻ | ◻ |
| Comments: ___________________________________________________________________ | | | | | |
| *A person of* ***melancholic*** *dominance in imbalance will tend to:* | | | | | |
|  | 1 | 2 | 3 | 4 | 5 |
| 2.9.16. Suffer from pathologies related to the nervous system, having an impact on the psyche or the body | ◻ | ◻ | ◻ | ◻ | ◻ |
| 2.9.17. Be depressed | ◻ | ◻ | ◻ | ◻ | ◻ |
| 2.9.18. Have digestive disorders such as bloating, gastritis, ulcers, alternating diarrhea/constipation, spasms, colic or dysbiosis | ◻ | ◻ | ◻ | ◻ | ◻ |
| 2.9.19. Express anxiety | ◻ | ◻ | ◻ | ◻ | ◻ |
| 2.9.20. Be prone to fungal infections and microbiota imbalances | ◻ | ◻ | ◻ | ◻ | ◻ |
| Comments: ___________________________________________________________________ | | | | | |

**2.10. OPEN QUESTIONS**

| **Open questions** | | | | | |
| --- | --- | --- | --- | --- | --- |
| 2.10.1. Do you have any comments or remarks on the work you have just done?  ***________________________________________________________________________________*** | | | | | |

The questionnaire is now complete. You can still go back and edit your answers if you wish to do so. If you have finished, you must click on the next page to validate your participation in this round of the questionnaire.

Thank you for taking part in the second round of the questionnaire for this study.

We are now at the halfway point of the four round questionnaire.

In a few weeks' time, we'll be sending you a link to the third round of the questionnaire. It will provide you with information on the overall results of the study at this stage, and will continue to explore the specific characteristics of each temperament.

Take care.

The Navi and UTS team

THIRD ROUND OF DELPHI QUESTIONNAIRE (3/4)

##

## **3.1. OBJECTIVES AND CONTENT OF THE THIRD QUESTIONNAIRE**

**Important notes:**

- Your participation in the third round is very valuable to us, whether or not you responded to the first and/or second rounds!
- For your answers to be taken into account, you must click on the final page to validate your participation in this round.

The aim is still to find a consensus that reflects the reality of the practice and teaching of this theory in the French-speaking naturopathic community.

1. For this new round, we need to collect your general information again.

2. You will be given statements to assess or characterize the dominance of the lymphatic, sanguine, choleric and melancholic temperaments.

These suggestions are quotations and reformulations based on data in the literature. We ask you to specify the extent to which they reflect your theoretical and/or practical experience.

You always have the opportunity to clarify your thoughts by adding remarks and comments in the “Comments” section.

Please note that after each round, we take your answers and comments into account to build the questionnaire for the next round and thus refine the consensus.

By using this link, you can return to the **participant information sheet** sent to you during recruitment. We remind you that **submitting the questionnaire online is an indication of your consent.**

## **Presentation of second-round results**

We thank you for your participation in the second round and are pleased to present some important results at this stage. Please read them before proceeding with the rest of the survey.

1. With regard to the concept of temperament, of the five proposals from the first round reinjected into the second round, two finally achieved consensus:

- The temperament is the activity of vital forces as it manifests in each individual.
- Temperament is inherently innate, but it also stems from the acquired.

The temperament quadrant (with the distribution of elements, qualities, and the principles of wetness and dryness) also won consensus, as shown below.


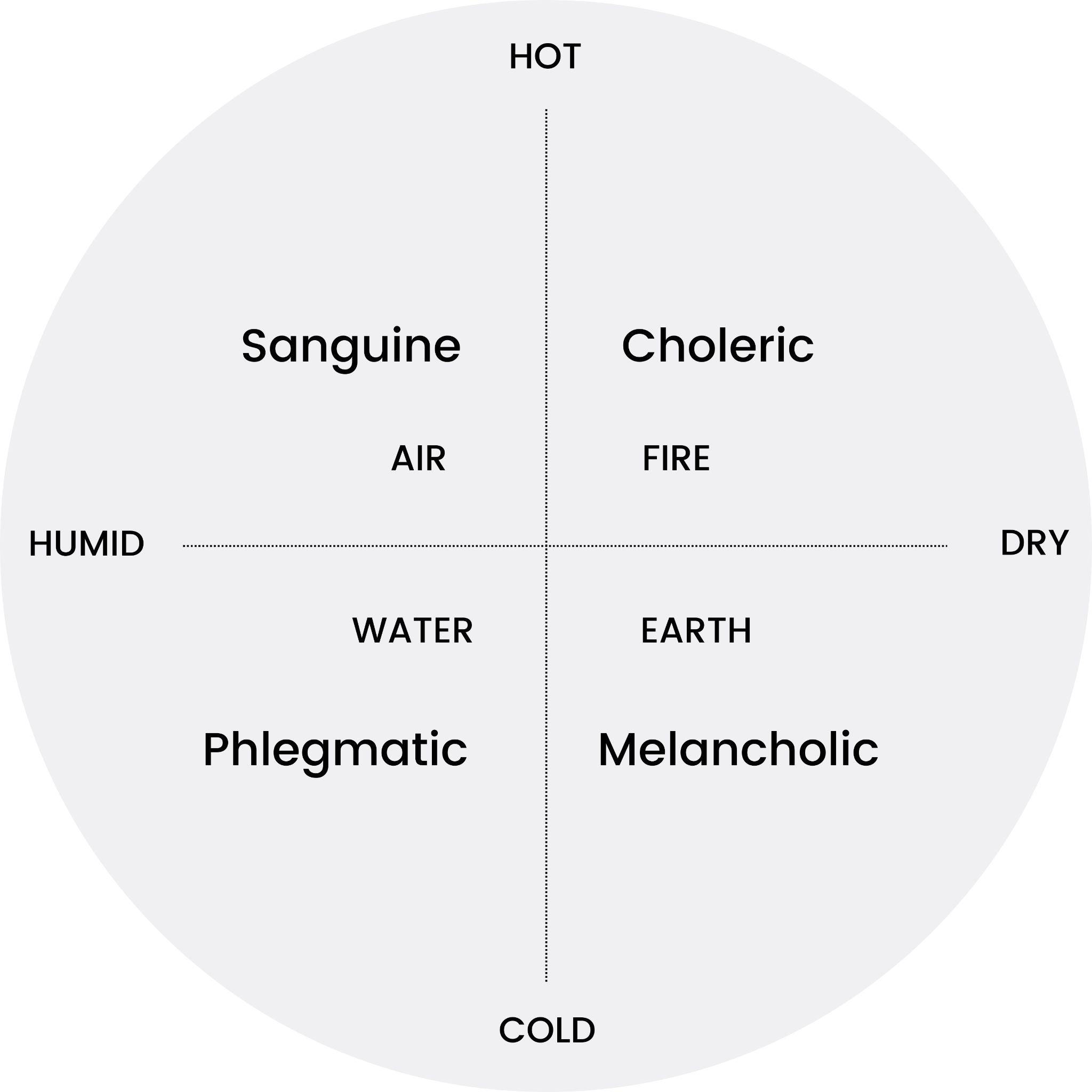


2. With regard to the **characteristics of each temperament**, 95 proposals reached consensus, 3 were rejected and 22 were forwarded to the next round for re-rating.

**In round 3**, which you are about to take part in, the 22 forwarded proposals are re-presented to you, and we invite you to vote on 120 new proposals.

##

## **3.2. ABOUT YOU**

Before we start, we need to gather some information about your practice again, to ensure that we collect a wide variety of perspectives on the subject, as well as to be able to relate these perspectives to the study results.

## **3.2.1. Which genre do you identify with?**

◻ Female

◻ Male

◻ Other

**3.2.2. What is your nationality? (several choices possible)**

◻ France

◻ Switzerland

◻ Belgium

◻ Luxembourg

◻ Canada

◻ Other :

**3.2.3. Which country do you work in? (several choices possible)**

◻ France

◻ Switzerland

◻ Belgium

◻ Luxembourg

◻ Canada

◻ Other :

**3.2.4. What is your professional activity related to the use of the Hippocratic temperament theory? (several choices possible)**

◻ Naturopathic practitioner (patient/client therapy)

◻ Researcher

◻ Teacher

◻ Author

◻ Practitioner in another discipline (phytotherapy, dietetics, etc.)

◻ Other :

**3.2.5. What type of institution do you work for? (several choices possible)**

◻ Private practice

◻ School / educational institution ...

◻ University

◻ Laboratory

◻ Research center

◻ Other :

**3.2.6. How many years have you been practicing with the Hippocratic temperament theory?**

◻ Less than 5 years

◻ From 5 to 10 years

◻ More than 10 years

**3.3. LITERARY RESOURCES ON TEMPERAMENTS**

| **Literary resources on temperaments** | | | | | |
| --- | --- | --- | --- | --- | --- |
| 24 literary sources of traditional and contemporary texts on the subject of Hippocratic temperaments were used to prepare this questionnaire. They are listed below: | | | | | |
| **Buckingham, R. M**. (2002). Extraversion, neuroticism and the four temperaments of antiquity: an investigation of physiological reactivity. Personality and Individual Differences, 2(2), 225-246.  **Carton, P**. (1961). Diagnostic et conduite des tempéraments. Librairie Le François.  **Carton, P.** (1984). Traité de médecine, d'alimentation et d'hygiène naturistes (4e éd.). A. Maloine & Fils.  **Dammeyer, J. & Zettler, I.** (2018). A Brief Historical Overview on Links Between Personality and Health. In C. Johansen (Ed.), Personality and Disease. Academic Press. 1-16.  **Garvelmann, F.** (2018). Konstitutionsmedizin. BACOPA.  **Gex, M.** (1949). Les classifications des tempéraments. Revue de Théologie et de Philosophie, 37(152), 147-162.  **Gunsburger, N.** (2017). Mon coach naturo : Mon programme sur mesure pour vivre en pleine santé. Eyrolles.  **Howart, E.** (1988). Mood differences between the four Galen personality types: choleric, sanguine, phlegmatic, melancholic. Personality and Individual Differences, 9(1), 173–175.  **Hutter, L.** (2017). Diagnostic humoral de la langue. Nhk Institut für integrative Naturheilkunde.  **Jouanna, J.** (2005). La théorie des quatre humeurs et des quatre tempéraments dans la tradition latine (Vindicien, Pseudo-Soranos) et une source grecque retrouvée. Revue des Études Grecques, 118, 138-167.  **Kieffer, D**. (2004). Guide personnel des bilans de santé : Encyclopédie naturopathique des tests morphologiques, psychologiques et biologiques de terrain (nouvelle éd.). Grancher.  **King, H. & Dasen, V.** (2008). La médecine dans l’antiquité grecque et romaine. Editions BHMS.  **Léaud-Zachoval, D.** (2017). Quatre clefs pour la santé : Lymphatique, sanguin, bilieux, nerveux, qui suis-je vraiment ? Editions Médicis  **Léaud-Zachoval, D.** (2021). Voyage au centre de la naturopathie. Hippocrate, 2(5), 17-25.  **Osborn, D.** (2007). Greek medicine. Greekmedicine.net  **Openpsychometrics.** (2019). OSPP Four Temperaments Test. Openpsychometrics.org.  **Raimann, C., Ganz, C., Garvelmann, F., Bertischi-Stahl, H., & Fehr Streule, R.** (2017). Grundlagen der Traditionellen Europäischen Naturheilkunde TEN. BACOPA.  **Rolfe, R.** (2002). The Four Temperaments. Marlowe & Compagny.  **Ruch, W.** (1992). Pavlov’s Types of Nervous System, Eysenck’s Typology and the Hippocrates-Galen Temperaments: an Empirical Examination of the Asserted Correspondence of Three Temperament Typologies. In Personality and individual Differences. 13(12), 1259-1271. Pergamon Press Ldt.  **Salmani Nodoushan, M. A.** (2011). Temperament as an indicator of language achievement. International Journal of Language Studies. 5. 33-52.  **Steiner, R, Laloux, M., Berthold-Andrea, H., Schad, W. & Smit, J**. (2014). L'énigme des tempéraments. Triades.  **Ternisien, L.** (2020). Naturopathie, le guide saison par saison. Flammarion.  **Vanopdenbosch, Y.** (2012). Les tempéraments : Outil de connaissance de soi et des autres. Amyris.  **Voutsinas, D.** (1961). Tempérament, constitution, caractère. Bulletin de psychologie, 15(197), 25-40. | | | | | |

**3.4. PSYCHO-EMOTIONAL DIMENSION**

| **Psycho-emotional dimension** | | | | | |
| --- | --- | --- | --- | --- | --- |
| *Mark how strongly you agree with the following statements regarding a person's psychological and emotional tendencies according to their dominant temperament.*  *1 Strongly Disagree, 2 Disagree, 3 Neutral, 4 Agree, 5 Strongly Agree.* | | | | | |
| *A person of* ***phlegmatic*** *dominance will tend to:* | | | | | |
|  | *1* | *2* | *3* | *4* | *5* |
| 3.4.1. Be emotionally stable by nature | ◻ | ◻ | ◻ | ◻ | ◻ |
| 3.4.2. Be introverted by nature | ◻ | ◻ | ◻ | ◻ | ◻ |
| 3.4.3. Be imaginative | ◻ | ◻ | ◻ | ◻ | ◻ |
| 3.4.4. Be sensitive to family fulfillment to find motivation | ◻ | ◻ | ◻ | ◻ | ◻ |
| 3.4.5. Be contemplative | ◻ | ◻ | ◻ | ◻ | ◻ |
| 3.4.6. Value safety | ◻ | ◻ | ◻ | ◻ | ◻ |
| 3.4.7. Be devoted to causes or people in need by nature | ◻ | ◻ | ◻ | ◻ | ◻ |
| 3.4.8. Enjoy being alone or in the company of a small circle | ◻ | ◻ | ◻ | ◻ | ◻ |
| 3.4.9. Be in his/her inner world | ◻ | ◻ | ◻ | ◻ | ◻ |
| Comments: ___________________________________________________________________ | | | | | |
| *A person of* ***sanguine*** *dominance will tend to:* | | | | | |
|  | *1* | *2* | *3* | *4* | *5* |
| 3.4.10. Be creative by nature | ◻ | ◻ | ◻ | ◻ | ◻ |
| 3.4.11. Move on quickly | ◻ | ◻ | ◻ | ◻ | ◻ |
| 3.4.12. Avoid conflict | ◻ | ◻ | ◻ | ◻ | ◻ |
| 3.4.13. Present emotional fluctuations | ◻ | ◻ | ◻ | ◻ | ◻ |
| 3.4.14. React quickly to stimuli | ◻ | ◻ | ◻ | ◻ | ◻ |
| 3.4.15. Explore the outside world rather than his/her inner life | ◻ | ◻ | ◻ | ◻ | ◻ |
| Comments: ___________________________________________________________________ | | | | | |
| *A person of* ***choleric*** *dominance will tend to:* | | | | | |
|  | *1* | *2* | *3* | *4* | *5* |
| 3.4.16. Be extroverted by nature | ◻ | ◻ | ◻ | ◻ | ◻ |
| 3.4.17. Experience changing emotions | ◻ | ◻ | ◻ | ◻ | ◻ |
| 3.4.18. Control his/her emotions | ◻ | ◻ | ◻ | ◻ | ◻ |
| 3.4.19. Have difficulty managing anger | ◻ | ◻ | ◻ | ◻ | ◻ |
| 3.4.20. Be sensitive by nature (without always showing it) | ◻ | ◻ | ◻ | ◻ | ◻ |
| 3.4.21. Be perseverant by nature | ◻ | ◻ | ◻ | ◻ | ◻ |
| 3.4.22. Live at maximum intensity and emotionality | ◻ | ◻ | ◻ | ◻ | ◻ |
| 3.4.23. Be responsible by nature | ◻ | ◻ | ◻ | ◻ | ◻ |
| 3.4.24. Be demanding by nature | ◻ | ◻ | ◻ | ◻ | ◻ |
| Comments: ___________________________________________________________________ | | | | | |
| *A person of* ***melancholic*** *dominance will tend to:* | | | | | |
|  | *1* | *2* | *3* | *4* | *5* |
| 3.4.25. Be pessimistic by nature | ◻ | ◻ | ◻ | ◻ | ◻ |
| 3.4.26. Reflect a lot | ◻ | ◻ | ◻ | ◻ | ◻ |
| 3.4.27. Be anxious by nature | ◻ | ◻ | ◻ | ◻ | ◻ |
| 3.4.28. Focus on the past | ◻ | ◻ | ◻ | ◻ | ◻ |
| 3.4.29. Feel internal nervousness or restlessness | ◻ | ◻ | ◻ | ◻ | ◻ |
| 3.4.30. Have great capacity for change | ◻ | ◻ | ◻ | ◻ | ◻ |
| 3.4.31. Be reserved by nature | ◻ | ◻ | ◻ | ◻ | ◻ |
| Comments: ___________________________________________________________________ | | | | | |

**3.5. BEHAVIORAL DIMENSION**

| **Behavioral dimension** | | | | | |
| --- | --- | --- | --- | --- | --- |
| *Mark how strongly you agree with the following statements regarding a person's behavioral tendencies according to their dominant temperament.*  *1 Strongly Disagree, 2 Disagree, 3 Neutral, 4 Agree, 5 Strongly Agree* | | | | | |
| *A person of* ***phlegmatic*** *dominance will tend to:* | | | | | |
|  | *1* | *2* | *3* | *4* | *5* |
| 3.5.1. Be highly adaptable | ◻ | ◻ | ◻ | ◻ | ◻ |
| 3.5.2. Work methodically and precisely | ◻ | ◻ | ◻ | ◻ | ◻ |
| 3.5.3. Be calm | ◻ | ◻ | ◻ | ◻ | ◻ |
| 3.5.4. Require stimulation and encouragement | ◻ | ◻ | ◻ | ◻ | ◻ |
| 3.5.5. Be reluctant to take part in sporting activities | ◻ | ◻ | ◻ | ◻ | ◻ |
| 3.5.6. Be persevering or enduring | ◻ | ◻ | ◻ | ◻ | ◻ |
| 3.5.7. Be present to things, events and people | ◻ | ◻ | ◻ | ◻ | ◻ |
| 3.5.8. Have a limp, weak handshake | ◻ | ◻ | ◻ | ◻ | ◻ |
| Comments: ___________________________________________________________________ | | | | | |
| *A person of* ***sanguine*** *dominance will tend to:* | | | | | |
|  | *1* | *2* | *3* | *4* | *5* |
| 3.5.9. Like outdoor professions | ◻ | ◻ | ◻ | ◻ | ◻ |
| 3.5.10. Have the need to be loved and admired | ◻ | ◻ | ◻ | ◻ | ◻ |
| 3.5.11. Have a lively spirit | ◻ | ◻ | ◻ | ◻ | ◻ |
| 3.5.12. Be generous | ◻ | ◻ | ◻ | ◻ | ◻ |
| 3.5.13. Lack perseverance | ◻ | ◻ | ◻ | ◻ | ◻ |
| 3.5.14. Be influenced by certain people | ◻ | ◻ | ◻ | ◻ | ◻ |
| 3.5.15. Have a strong handshake | ◻ | ◻ | ◻ | ◻ | ◻ |
| Comments: ___________________________________________________________________ | | | | | |
| *A person of* ***choleric*** *dominance will tend to:* | | | | | |
|  | *1* | *2* | *3* | *4* | *5* |
| 3.5.16. Be authoritarian and domineering, when out of balance | ◻ | ◻ | ◻ | ◻ | ◻ |
| 3.5.17. Be comfortable in leadership roles | ◻ | ◻ | ◻ | ◻ | ◻ |
| 3.5.18. Have ambition | ◻ | ◻ | ◻ | ◻ | ◻ |
| 3.5.19. Give importance to following rules and justice | ◻ | ◻ | ◻ | ◻ | ◻ |
| 3.5.20. Be impatient | ◻ | ◻ | ◻ | ◻ | ◻ |
| 3.5.21. Give a firm handshake | ◻ | ◻ | ◻ | ◻ | ◻ |
| Comments: ___________________________________________________________________ | | | | | |
| *A person of* ***melancholic*** *dominance will tend to:* | | | | | |
|  | *1* | *2* | *3* | *4* | *5* |
| 3.5.22. Have jerky gestures | ◻ | ◻ | ◻ | ◻ | ◻ |
| 3.5.23. Be deprived | ◻ | ◻ | ◻ | ◻ | ◻ |
| 3.5.24. Divert sexual energy to other activities | ◻ | ◻ | ◻ | ◻ | ◻ |
| 3.5.25. Do little physical exercise | ◻ | ◻ | ◻ | ◻ | ◻ |
| 3.5.26. Have a quick and irregular rhythm in their speech | ◻ | ◻ | ◻ | ◻ | ◻ |
| 3.5.27. Deliver a short, firm and rapid handshake | ◻ | ◻ | ◻ | ◻ | ◻ |
| Comments: ___________________________________________________________________ | | | | | |

**3.6. PHYSIOLOGICAL DIMENSION**

| **Physiological dimension** | | | | | |
| --- | --- | --- | --- | --- | --- |
| *Mark how strongly you agree with the following statements regarding a person's physiological tendencies (in the sense of normal body functions and reactions) according to their dominant temperament.*  *1 Strongly Disagree, 2 Disagree, 3 Neutral, 4 Agree, 5 Strongly Agree.* | | | | | |
| *A person of* ***phlegmatic*** *dominance will tend to:* | | | | | |
|  | *1* | *2* | *3* | *4* | *5* |
| 3.6.1. Lack energy | ◻ | ◻ | ◻ | ◻ | ◻ |
| 3.6.2. Have a rather strong appetite | ◻ | ◻ | ◻ | ◻ | ◻ |
| 3.6.3. Be sensitive to cold temperatures | ◻ | ◻ | ◻ | ◻ | ◻ |
| 3.6.4. Have a very slow metabolism | ◻ | ◻ | ◻ | ◻ | ◻ |
| 3.6.5. Recover slowly from physical effort | ◻ | ◻ | ◻ | ◻ | ◻ |
| 3.6.6. Have a poor blood and lymph circulation | ◻ | ◻ | ◻ | ◻ | ◻ |
| 3.6.7. Have high stamina | ◻ | ◻ | ◻ | ◻ | ◻ |
| 3.6.8. Have no digestive fire | ◻ | ◻ | ◻ | ◻ | ◻ |
| 3.6.9. Use touch as a dominant sense | ◻ | ◻ | ◻ | ◻ | ◻ |
| Comments: ___________________________________________________________________ | | | | | |
| *A person of* ***sanguine*** *dominance will tend to:* | | | | | |
|  | *1* | *2* | *3* | *4* | *5* |
| 3.6.10. Produce energy efficiently, even throughout the day | ◻ | ◻ | ◻ | ◻ | ◻ |
| 3.6.11. Have a fragile cardiovascular system | ◻ | ◻ | ◻ | ◻ | ◻ |
| 3.6.12. Recover well from physical exertion or mental stress | ◻ | ◻ | ◻ | ◻ | ◻ |
| 3.6.13. Sweat profusely | ◻ | ◻ | ◻ | ◻ | ◻ |
| 3.6.14. Have a catabolic metabolism (breakdown, elimination and energy production) | ◻ | ◻ | ◻ | ◻ | ◻ |
| 3.6.15. Have a dominant sense of taste and smell | ◻ | ◻ | ◻ | ◻ | ◻ |
| Comments: ___________________________________________________________________ | | | | | |
| *A person of* ***choleric*** *dominance will tend to:* | | | | | |
|  | *1* | *2* | *3* | *4* | *5* |
| 3.6.16. Have a recuperative sleep | ◻ | ◻ | ◻ | ◻ | ◻ |
| 3.6.17. Digest quickly and well | ◻ | ◻ | ◻ | ◻ | ◻ |
| 3.6.18. Have a high level of activity and movement | ◻ | ◻ | ◻ | ◻ | ◻ |
| 3.6.19. Have a hepato-biliary weakness | ◻ | ◻ | ◻ | ◻ | ◻ |
| 3.6.20. Easily present with accelerated adrenal glands | ◻ | ◻ | ◻ | ◻ | ◻ |
| 3.6.21. Have metabolic acidity (acid waste deposits in joints, gastric hyperacidity, etc.) | ◻ | ◻ | ◻ | ◻ | ◻ |
| 3.6.22. Have a rather strong digestive fire | ◻ | ◻ | ◻ | ◻ | ◻ |
| 3.5.23. Use sight as dominant sense | ◻ | ◻ | ◻ | ◻ | ◻ |
| Comments: ___________________________________________________________________ | | | | | |
| *A person of* ***melancholic*** *dominance will tend to:* | | | | | |
|  | *1* | *2* | *3* | *4* | *5* |
| 3.6.24. Lack energy | ◻ | ◻ | ◻ | ◻ | ◻ |
| 3.6.25. Eat little and often | ◻ | ◻ | ◻ | ◻ | ◻ |
| 3.6.26. Sweat little | ◻ | ◻ | ◻ | ◻ | ◻ |
| 3.6.27. Be sensitive at the neuroendocrine level | ◻ | ◻ | ◻ | ◻ | ◻ |
| 3.6.28. Have their energy concentrated at the level of the brain | ◻ | ◻ | ◻ | ◻ | ◻ |
| 3.6.29. Have a fluctuating energy | ◻ | ◻ | ◻ | ◻ | ◻ |
| 3.6.30. Use hearing as a dominant sense | ◻ | ◻ | ◻ | ◻ | ◻ |
| Comments: ___________________________________________________________________ | | | | | |

**3.7. MORPHOLOGICAL DIMENSION**

| **Morphological dimension** | | | | | |
| --- | --- | --- | --- | --- | --- |
| *Mark how strongly you agree with the following statements regarding a person's physical traits according to their dominant temperament.*  *1 Strongly Disagree, 2 Disagree, 3 Neutral, 4 Agree, 5 Strongly Agree.* | | | | | |
| *A person of* ***phlegmatic*** *dominance will tend to:* | | | | | |
|  | *1* | *2* | *3* | *4* | *5* |
| 3.7.1. Have a brevilineal or cobby type build (ie. with a rather long trunk and rather short limbs) | ◻ | ◻ | ◻ | ◻ | ◻ |
| 3.7.2. Have a rounded face | ◻ | ◻ | ◻ | ◻ | ◻ |
| 3.7.3. Have a small nose | ◻ | ◻ | ◻ | ◻ | ◻ |
| 3.7.4. Have a moist tongue that may be swollen or edematous | ◻ | ◻ | ◻ | ◻ | ◻ |
| 3.7.5. Have a pale tongue | ◻ | ◻ | ◻ | ◻ | ◻ |
| 3.7.6. Have square fingers | ◻ | ◻ | ◻ | ◻ | ◻ |
| 3.7.7. Have fingers that are shorter than the palm | ◻ | ◻ | ◻ | ◻ | ◻ |
| Comments: ___________________________________________________________________ | | | | | |
| *A person of* ***sanguine*** *dominance will tend to:* | | | | | |
|  | *1* | *2* | *3* | *4* | *5* |
| 3.7.8. Have more developed mid-face (the part between the top of the eyebrows and the line marking the lower edge of the nose and cheekbones) | ◻ | ◻ | ◻ | ◻ | ◻ |
| 3.7.9. Have a massive nose | ◻ | ◻ | ◻ | ◻ | ◻ |
| 3.7.10. Have a large, sometimes swollen tongue | ◻ | ◻ | ◻ | ◻ | ◻ |
| 3.7.11. Have pink to red, sometimes even purple tongue | ◻ | ◻ | ◻ | ◻ | ◻ |
| 3.7.12. Have large, fleshy hands | ◻ | ◻ | ◻ | ◻ | ◻ |
| 3.7.13. Have short, strong fingers | ◻ | ◻ | ◻ | ◻ | ◻ |
| Comments: ___________________________________________________________________ | | | | | |
| *A person of* ***choleric*** *dominance will tend to:* | | | | | |
|  | *1* | *2* | *3* | *4* | *5* |
| 3.7.14. Have a square or rectangular face shape | ◻ | ◻ | ◻ | ◻ | ◻ |
| 3.7.15. Have a prominent, bony or angular nose | ◻ | ◻ | ◻ | ◻ | ◻ |
| 3.7.16. Have a red tongue or tongue with red spots on the surface | ◻ | ◻ | ◻ | ◻ | ◻ |
| 3.7.17. Have a dry tongue with grooves or fissures | ◻ | ◻ | ◻ | ◻ | ◻ |
| 3.7.18. Have a square or rectangular hand shape | ◻ | ◻ | ◻ | ◻ | ◻ |
| 3.7.19. Have fingers equal to or longer than the palms of the hand | ◻ | ◻ | ◻ | ◻ | ◻ |
| Comments: ___________________________________________________________________ | | | | | |
| *A person of* ***melancholic*** *dominance will tend to:* | | | | | |
|  | *1* | *2* | *3* | *4* | *5* |
| 3.7.20. Have a retracted, triangular face with an inverted point | ◻ | ◻ | ◻ | ◻ | ◻ |
| 3.7.21. Have a fine, pointed nose | ◻ | ◻ | ◻ | ◻ | ◻ |
| 3.7.22. Have a small, firm, pointed tongue | ◻ | ◻ | ◻ | ◻ | ◻ |
| 3.7.23. Have a pale or bluish tongue | ◻ | ◻ | ◻ | ◻ | ◻ |
| 3.7.24. Have hands that are increasingly slender from palm to fingertips | ◻ | ◻ | ◻ | ◻ | ◻ |
| 3.7.25. Have long and slender fingers with enlarged joints that may be hypermobile. | ◻ | ◻ | ◻ | ◻ | ◻ |
| Comments: ___________________________________________________________________ | | | | | |

**3.8. SUSCEPTIBILITY TO DISEASES TO DISEASES**

| **Susceptibility to diseases** | | | | | |
| --- | --- | --- | --- | --- | --- |
| *Mark how strongly you agree with the following statements regarding a person's vulnerability to disease and imbalance, according to their dominant temperament.*  *1 Strongly Disagree, 2 Disagree, 3 Neutral, 4 Agree, 5 Strongly Agree.* | | | | | |
| *A person of* ***phlegmatic*** *dominance in imbalance will tend to:* | | | | | |
|  | 1 | 2 | 3 | 4 | 5 |
| 3.8.1. Express ENT (Ear-Nose-Throat) pathologies | ◻ | ◻ | ◻ | ◻ | ◻ |
| 3.8.2. Be addicted to substances such as cigarettes, light beer, sweet wines or excessive help to others | ◻ | ◻ | ◻ | ◻ | ◻ |
| 3.8.3. Suffer from constipation | ◻ | ◻ | ◻ | ◻ | ◻ |
| 3.8.4. Gain weight or be overweight | ◻ | ◻ | ◻ | ◻ | ◻ |
| 3.8.5. Be prone to chronic fatigue | ◻ | ◻ | ◻ | ◻ | ◻ |
| 3.8.6. Being depressed | ◻ | ◻ | ◻ | ◻ | ◻ |
| 3.8.7. Have lymph node pathologies | ◻ | ◻ | ◻ | ◻ | ◻ |
| 3.8.8. Respond to intense, long-lasting stimuli as part of therapeutic support | ◻ | ◻ | ◻ | ◻ | ◻ |
| Comments: ___________________________________________________________________ | | | | | |
| *A person of* ***sanguine*** *dominance in imbalance will tend to:* | | | | | |
|  | 1 | 2 | 3 | 4 | 5 |
| 3.8.9. Develop respiratory fragility, such as pneumonia or asthma | ◻ | ◻ | ◻ | ◻ | ◻ |
| 3.8.10. Easily have fever | ◻ | ◻ | ◻ | ◻ | ◻ |
| 3.8.11. Suffer from liver failure | ◻ | ◻ | ◻ | ◻ | ◻ |
| 3.8.12. Have fragile mucous membranes, particularly in the digestive, respiratory and genitourinary tracts | ◻ | ◻ | ◻ | ◻ | ◻ |
| 3.8.13. Heal quickly | ◻ | ◻ | ◻ | ◻ | ◻ |
| 3.8.14. Have mild allergies in childhood, replaced by excess weight in adulthood | ◻ | ◻ | ◻ | ◻ | ◻ |
| 3.8.15. Respond quickly to small stimuli, as part of therapeutic support | ◻ | ◻ | ◻ | ◻ | ◻ |
| Comments: ___________________________________________________________________ | | | | | |
| *A person of* ***choleric*** *dominance in imbalance will tend to:* | | | | | |
|  | 1 | 2 | 3 | 4 | 5 |
| 3.8.16. Have gallstone formation | ◻ | ◻ | ◻ | ◻ | ◻ |
| 3.8.17. Accumulate cholesterol | ◻ | ◻ | ◻ | ◻ | ◻ |
| 3.8.18. Poor immunity | ◻ | ◻ | ◻ | ◻ | ◻ |
| 3.8.19. Suffer from neurovegetative dystonia (a group of nervous, emotional and digestive disorders linked to deregulation of the autonomic nervous system) | ◻ | ◻ | ◻ | ◻ | ◻ |
| 3.8.20. Present violent symptoms at the onset of illness due to lack of energy or moisture reserves | ◻ | ◻ | ◻ | ◻ | ◻ |
| 3.8.21. Suffer from inflammation due to insufficient waste elimination when lacking physical activity | ◻ | ◻ | ◻ | ◻ | ◻ |
| Comments: ___________________________________________________________________ | | | | | |
| *A person of* ***melancholic*** *dominance in imbalance will tend to:* | | | | | |
|  | 1 | 2 | 3 | 4 | 5 |
| 3.8.22. Be predisposed to fungal infections and microbiota imbalances | ◻ | ◻ | ◻ | ◻ | ◻ |
| 3.8.23. Have addictions to substances such as wine, beer, sugar and chocolate, and also coffee and nicotine | ◻ | ◻ | ◻ | ◻ | ◻ |
| 3.8.24. Have mineral deficiencies | ◻ | ◻ | ◻ | ◻ | ◻ |
| 3.8.25. Be exposed to pathologies relating to acidosis | ◻ | ◻ | ◻ | ◻ | ◻ |
| 3.8.26. Have joints pathologies such as lordosis, osteoarthritis or scoliosis | ◻ | ◻ | ◻ | ◻ | ◻ |
| 3.8.27. Have migraines | ◻ | ◻ | ◻ | ◻ | ◻ |
| 3.8.28. Have serious or degenerative auto-immune, neuro-endocrine or cancerous diseases | ◻ | ◻ | ◻ | ◻ | ◻ |
| 3.8.29. Suffer from eating disorders, such as lack of appetite, or in more serious case, anorexia or alternating bulimia-anorexia | ◻ | ◻ | ◻ | ◻ | ◻ |
| Comments: ___________________________________________________________________ | | | | | |

**3.9. Final comments**

| **Open questions** | | | | | |
| --- | --- | --- | --- | --- | --- |
| 3.9.1. Do you have any comments or remarks on the questionnaire you have just completed?  ***_______________________________________________________________________________*** | | | | | |

The questionnaire is now complete. You can still go back and edit your answers if you wish. If you have finished, you must click on the next page to validate your participation in this round of the questionnaire.

Thank you for taking part in the third round of the questionnaire for this study.

We're now three-quarters of the way through the four round questionnaire.

In a few weeks' time, we'll be sending you a link to the fourth round of the questionnaire. It will provide you with information on the overall results of the study at this stage, and will finish exploring the characteristics specific to each temperament.

Take care.

The Navi and UTS team.

FOURTH ROUND OF DELPHI QUESTIONNAIRE (4/4)

## **4.1. OBJECTIVES AND CONTENT OF THE FOURTH AND LAST QUESTIONNAIRE**

**Important notes:**

- Your participation in the fourth round is very valuable to us, whether or not you participated in the first, second and/or third rounds!
- For your answers to be taken into account, you must click on the final page to validate your participation in this round.

The aim is still to find a consensus that reflects the reality of the practice and teaching of this theory in the French-speaking naturopathic community.

1. For this fourth and final round, we need to collect your general information again.

2. The aim is still to assess or characterize the dominance of the lymphatic, sanguine, choleric and melancholic temperaments.

These proposals are quotations and reformulations based on data in the literature. We ask you to specify the extent to which they reflect your theoretical and/or practical experience.

You always have the opportunity to clarify your thoughts by adding remarks and comments in the “Comments” section.

By using this link, you can consult the participant information sheet sent to you during recruitment. We remind you that submitting the questionnaire online is an indication of your consent.

## **Presentation of third-round results**

We thank you for your participation in the third round and are pleased to present you some important results at this stage. Please read them before proceeding with the rest of the study.

With regard to **the characteristics of each temperament,** 102 proposals were accepted by consensus, 7 were rejected and 33 were undecided.

**In round 4, which you are about to take part in, the proposals that were undecided in round 3 are re-presented to you, sometimes in a slightly different form.**

**It's normal for a temperament to be sometimes missing in a section, if all proposals have been accepted in round 3 (e.g. choleric in the behavioral section).**

## **4.2. ABOUT YOU**

Before we start, we need to gather some information about your practice again, to ensure that we collect a wide variety of perspectives on the subject, as well as to be able to relate these perspectives to the study results.

## **4.2.1. Which genre do you identify with?**

◻ Female

◻ Male

◻ Other

**4.2.2. What is your nationality? (several choices possible)**

◻ France

◻ Switzerland

◻ Belgium

◻ Luxembourg

◻ Canada

◻ Other :

**4.2.3. Which country do you work in? (several choices possible)**

◻ France

◻ Switzerland

◻ Belgium

◻ Luxembourg

◻ Canada

◻ Other :

**4.2.4. What is your professional activity related to the use of the Hippocratic temperament theory? (several choices possible)**

◻ Naturopathic practitioner (patient/client therapy)

◻ Researcher

◻ Teacher

◻ Author

◻ Practitioner in another discipline (phytotherapy, dietetics, etc.)

◻ Other :

**4.2.5. What type of institution do you work for? (several choices possible)**

◻ Private practice

◻ School / educational institution ...

◻ University

◻ Laboratory

◻ Research center

◻ Other :

**4.2.6. How many years have you been practicing with the Hippocratic temperament theory?**

◻ Less than 5 years

◻ From 5 to 10 years

◻ More than 10 years

**4.3. LITERARY RESOURCES ON TEMPERAMENTS**

| **Literary resources on temperaments** | | | | | |
| --- | --- | --- | --- | --- | --- |
| 24 literary sources of traditional and contemporary texts on the subject of Hippocratic temperaments were used to prepare this questionnaire. They are listed below: | | | | | |
| **Buckingham, R. M**. (2002). Extraversion, neuroticism and the four temperaments of antiquity: an investigation of physiological reactivity. Personality and Individual Differences, 2(2), 225-246.  **Carton, P**. (1961). Diagnostic et conduite des tempéraments. Librairie Le François.  **Carton, P.** (1984). Traité de médecine, d'alimentation et d'hygiène naturistes (4e éd.). A. Maloine & Fils.  **Dammeyer, J. & Zettler, I.** (2018). A Brief Historical Overview on Links Between Personality and Health. In C. Johansen (Ed.), Personality and Disease. Academic Press. 1-16.  **Garvelmann, F.** (2018). Konstitutionsmedizin. BACOPA.  **Gex, M.** (1949). Les classifications des tempéraments. Revue de Théologie et de Philosophie, 37(152), 147-162.  **Gunsburger, N.** (2017). Mon coach naturo : Mon programme sur mesure pour vivre en pleine santé. Eyrolles.  **Howart, E.** (1988). Mood differences between the four Galen personality types: choleric, sanguine, phlegmatic, melancholic. Personality and Individual Differences, 9(1), 173–175.  **Hutter, L.** (2017). Diagnostic humoral de la langue. Nhk Institut für integrative Naturheilkunde.  **Jouanna, J.** (2005). La théorie des quatre humeurs et des quatre tempéraments dans la tradition latine (Vindicien, Pseudo-Soranos) et une source grecque retrouvée. Revue des Études Grecques, 118, 138-167.  **Kieffer, D**. (2004). Guide personnel des bilans de santé : Encyclopédie naturopathique des tests morphologiques, psychologiques et biologiques de terrain (nouvelle éd.). Grancher.  **King, H. & Dasen, V.** (2008). La médecine dans l’antiquité grecque et romaine. Editions BHMS.  **Léaud-Zachoval, D.** (2017). Quatre clefs pour la santé : Lymphatique, sanguin, bilieux, nerveux, qui suis-je vraiment ? Editions Médicis  **Léaud-Zachoval, D.** (2021). Voyage au centre de la naturopathie. Hippocrate, 2(5), 17-25.  **Osborn, D.** (2007). Greek medicine. Greekmedicine.net  **Openpsychometrics.** (2019). OSPP Four Temperaments Test. Openpsychometrics.org.  **Raimann, C., Ganz, C., Garvelmann, F., Bertischi-Stahl, H., & Fehr Streule, R.** (2017). Grundlagen der Traditionellen Europäischen Naturheilkunde TEN. BACOPA.  **Rolfe, R.** (2002). The Four Temperaments. Marlowe & Compagny.  **Ruch, W.** (1992). Pavlov’s Types of Nervous System, Eysenck’s Typology and the Hippocrates-Galen Temperaments: an Empirical Examination of the Asserted Correspondence of Three Temperament Typologies. In Personality and individual Differences. 13(12), 1259-1271. Pergamon Press Ldt.  **Salmani Nodoushan, M. A.** (2011). Temperament as an indicator of language achievement. International Journal of Language Studies. 5. 33-52.  **Steiner, R, Laloux, M., Berthold-Andrea, H., Schad, W. & Smit, J**. (2014). L'énigme des tempéraments. Triades.  **Ternisien, L.** (2020). Naturopathie, le guide saison par saison. Flammarion.  **Vanopdenbosch, Y.** (2012). Les tempéraments : Outil de connaissance de soi et des autres. Amyris.  **Voutsinas, D.** (1961). Tempérament, constitution, caractère. Bulletin de psychologie, 15(197), 25-40. | | | | | |

**4.4. PSYCHO-EMOTIONAL DIMENSION**

| **Psycho-emotional dimension** | | | | | |
| --- | --- | --- | --- | --- | --- |
| *Mark how strongly you agree with the following statements regarding a person's psychological and emotional tendencies according to their dominant temperament.*  *1 Strongly Disagree, 2 Disagree, 3 Neutral, 4 Agree, 5 Strongly Agree.* | | | | | |
| *A person of* ***phlegmatic*** *dominance will tend to:* | | | | | |
|  | *1* | *2* | *3* | *4* | *5* |
| 4.4.1. Be emotionally stable by nature | ◻ | ◻ | ◻ | ◻ | ◻ |
| 4.4.2. Be imaginative | ◻ | ◻ | ◻ | ◻ | ◻ |
| 4.4.3. Enjoy solitude | ◻ | ◻ | ◻ | ◻ | ◻ |
| 4.4.4. Enjoy one-to-one discussions | ◻ | ◻ | ◻ | ◻ | ◻ |
| Comments: ___________________________________________________________________ | | | | | |
| *A person of* ***sanguine*** *dominance will tend to:* | | | | | |
|  | *1* | *2* | *3* | *4* | *5* |
| 4.4.5. Be creative by nature | ◻ | ◻ | ◻ | ◻ | ◻ |
| Comments: ___________________________________________________________________ | | | | | |
| *A person of* ***choleric*** *dominance will tend to:* | | | | | |
|  | *1* | *2* | *3* | *4* | *5* |
| 4.4.6. Control his/her emotions | ◻ | ◻ | ◻ | ◻ | ◻ |
| Comments: ___________________________________________________________________ | | | | | |
| *A person of* ***melancholic*** *dominance will tend to:* | | | | | |
|  | *1* | *2* | *3* | *4* | *5* |
| 4.4.7. Focus on the past | ◻ | ◻ | ◻ | ◻ | ◻ |
| Comments: ___________________________________________________________________ | | | | | |

**4.5. BEHAVIORAL DIMENSION**

| **Behavioral dimension** | | | | | |
| --- | --- | --- | --- | --- | --- |
| *Mark how strongly you agree with the following statements regarding a person's behavioral tendencies according to their dominant temperament.*  *1 Strongly Disagree, 2 Disagree, 3 Neutral, 4 Agree, 5 Strongly Agree* | | | | | |
| *A person of* ***phlegmatic*** *dominance will tend to:* | | | | | |
|  | *1* | *2* | *3* | *4* | *5* |
| 4.5.1. Be highly adaptable | ◻ | ◻ | ◻ | ◻ | ◻ |
| 4.5.2 Work methodically | ◻ | ◻ | ◻ | ◻ | ◻ |
| 4.5.3. Work precisely | ◻ | ◻ | ◻ | ◻ | ◻ |
| 3.5.4. Be present to things, events and people, thanks to his/her ability to anchor | ◻ | ◻ | ◻ | ◻ | ◻ |
| Comments: ___________________________________________________________________ | | | | | |
| *A person of* ***sanguine*** *dominance will tend to:* | | | | | |
|  | *1* | *2* | *3* | *4* | *5* |
| 4.5.5. Lack perseverance | ◻ | ◻ | ◻ | ◻ | ◻ |
| 4.5.6. Be influenced by certain people | ◻ | ◻ | ◻ | ◻ | ◻ |
| Comments: ___________________________________________________________________ | | | | | |
| *A person of* ***melancholic*** *dominance will tend to:* | | | | | |
|  | *1* | *2* | *3* | *4* | *5* |
| 4.5.7. Divert sexual energy to other activities | ◻ | ◻ | ◻ | ◻ | ◻ |
| 4.5.8. Do little physical exercise | ◻ | ◻ | ◻ | ◻ | ◻ |
| Comments: ___________________________________________________________________ | | | | | |

**4.6. PHYSIOLOGICAL DIMENSION**

| **Physiological dimension** | | | | | |
| --- | --- | --- | --- | --- | --- |
| *Mark how strongly you agree with the following statements regarding a person's physiological tendencies (in the sense of normal body functions and reactions) according to their dominant temperament.*  *1 Strongly Disagree, 2 Disagree, 3 Neutral, 4 Agree, 5 Strongly Agree.* | | | | | |
| *A person of* ***phlegmatic*** *dominance will tend to:* | | | | | |
|  | *1* | *2* | *3* | *4* | *5* |
| 4.6.1. Be sensitive to cold temperatures | ◻ | ◻ | ◻ | ◻ | ◻ |
| 4.6.2. Use touch as a dominant sense | ◻ | ◻ | ◻ | ◻ | ◻ |
| Comments: ___________________________________________________________________ | | | | | |
| *A person of* ***sanguine*** *dominance will tend to:* | | | | | |
|  | *1* | *2* | *3* | *4* | *5* |
| 4.6.3. Have a dominant sense of taste | ◻ | ◻ | ◻ | ◻ | ◻ |
| 4.6.4. Have a dominant sense of smell | ◻ | ◻ | ◻ | ◻ | ◻ |
| Comments: ___________________________________________________________________ | | | | | |
| *A person of* ***choleric*** *dominance will tend to:* | | | | | |
|  | *1* | *2* | *3* | *4* | *5* |
| 4.6.6. Digest quickly and well | ◻ | ◻ | ◻ | ◻ | ◻ |
| 4.6.7. Digest well | ◻ | ◻ | ◻ | ◻ | ◻ |
| 4.6.8. Use sight as dominant sense | ◻ | ◻ | ◻ | ◻ | ◻ |
| Comments: ___________________________________________________________________ | | | | | |

**4.7. MORPHOLOGICAL DIMENSION**

| **Morphological dimension** | | | | | |
| --- | --- | --- | --- | --- | --- |
| *Mark how strongly you agree with the following statements regarding a person's physical traits according to their dominant temperament.*  *1 Strongly Disagree, 2 Disagree, 3 Neutral, 4 Agree, 5 Strongly Agree.* | | | | | |
| *A person of* ***phlegmatic*** *dominance will tend to:* | | | | | |
|  | *1* | *2* | *3* | *4* | *5* |
| 4.7.1. Have a small nose | ◻ | ◻ | ◻ | ◻ | ◻ |
| 4.7.2. Have a pale tongue | ◻ | ◻ | ◻ | ◻ | ◻ |
| 4.7.3. Have square fingers | ◻ | ◻ | ◻ | ◻ | ◻ |
| Comments: ___________________________________________________________________ | | | | | |
| *A person of* ***choleric*** *dominance will tend to:* | | | | | |
|  | *1* | *2* | *3* | *4* | *5* |
| 4.7.4. Have a red tongue | ◻ | ◻ | ◻ | ◻ | ◻ |
| 4.7.5. Have a tongue with red spots on the surface | ◻ | ◻ | ◻ | ◻ | ◻ |
| 4.7.6. Have a dry tongue | ◻ | ◻ | ◻ | ◻ | ◻ |
| 4.7.7. Have a tongue with grooves or cracks | ◻ | ◻ | ◻ | ◻ | ◻ |
| Comments: ___________________________________________________________________ | | | | | |
| *A person of* ***melancholic*** *dominance will tend to:* | | | | | |
|  | *1* | *2* | *3* | *4* | *5* |
| 4.7.8. Have a pale tongue | ◻ | ◻ | ◻ | ◻ | ◻ |
| 4.7.9. Have a bluish tongue | ◻ | ◻ | ◻ | ◻ | ◻ |
| Comments: ___________________________________________________________________ | | | | | |

**4.8. SUSCEPTIBILITY TO DISEASES TO DISEASES**

| **Susceptibility to diseases** | | | | | |
| --- | --- | --- | --- | --- | --- |
| *Mark how strongly you agree with the following statements regarding a person's vulnerability to disease and imbalance, according to their dominant temperament.*  *1 Strongly Disagree, 2 Disagree, 3 Neutral, 4 Agree, 5 Strongly Agree.* | | | | | |
| *A person of* ***phlegmatic*** *dominance in imbalance will tend to:* | | | | | |
|  | 1 | 2 | 3 | 4 | 5 |
| 4.8.1. Be addicted to substances such as cigarettes | ◻ | ◻ | ◻ | ◻ | ◻ |
| 4.8.2. Be addicted to substances such as light beer or sweet wines | ◻ | ◻ | ◻ | ◻ | ◻ |
| 4.8.3. Be addicted to excessively help others | ◻ | ◻ | ◻ | ◻ | ◻ |
| 4.8.4. Be depressed | ◻ | ◻ | ◻ | ◻ | ◻ |
| 4.8.5. Respond to intense, long-lasting stimuli as part of therapeutic support | ◻ | ◻ | ◻ | ◻ | ◻ |
| Comments: ___________________________________________________________________ | | | | | |
| *A person of* ***sanguine*** *dominance in imbalance will tend to:* | | | | | |
|  | 1 | 2 | 3 | 4 | 5 |
| 4.8.6. Have a fragile respiratory system | ◻ | ◻ | ◻ | ◻ | ◻ |
| 4.8.7.Suffer from liver failure | ◻ | ◻ | ◻ | ◻ | ◻ |
| 4.8.8. Have fragil digestive mucosa | ◻ | ◻ | ◻ | ◻ | ◻ |
| 4.8.9. Have fragile respiratory mucosa | ◻ | ◻ | ◻ | ◻ | ◻ |
| 4.8.10. Have fragile genito-urinary mucosa | ◻ | ◻ | ◻ | ◻ | ◻ |
| 4.8.11. Have mild allergies in childhood, replaced by excess weight in adulthood | ◻ | ◻ | ◻ | ◻ | ◻ |
| Comments: ___________________________________________________________________ | | | | | |
| *A person of* ***choleric*** *dominance in imbalance will tend to:* | | | | | |
|  | 1 | 2 | 3 | 4 | 5 |
| 4.8.12. Suffer from neurovegetative dystonia (a group of nervous, emotional and digestive disorders linked to deregulation of the autonomic nervous system) | ◻ | ◻ | ◻ | ◻ | ◻ |
| 4.8.13. Present violent symptoms at the onset of illness due to lack of energy or moisture reserves | ◻ | ◻ | ◻ | ◻ | ◻ |
| Comments: ___________________________________________________________________ | | | | | |

**4.9. Final comments**

| **Open questions** | | | | | |
| --- | --- | --- | --- | --- | --- |
| 4.9.1. Do you have any comments or remarks on the questionnaire you have just completed?  *_______________________________________________________________________________* | | | | | |

The questionnaire is now complete. You can still go back and edit your answers if you wish. If you have finished, you must click on the next page to validate your participation in this round of the questionnaire.

Thank you for taking part in the fourth round of the questionnaire for this study.

We're now at the end of the four round questionnaire.

In the coming months, we will keep you informed of the results of the study and the progress of this project.

Thank you very much for taking part in this study!

Take care.

The Navi and UTS team.
