# Supplementary material for: Establishing a consensus on the clinical assessment of Hippocratic temperaments in the French-speaking naturopathic community: a Delphi study
Source: BMC Complement Med Ther. 2026 Mar 29;26:173. doi: 10.1186/s12906-026-05353-y (PMC13151160; doi:10.1186/s12906-026-05353-y)
Supplement: Supplementary file 2 — Supplementary Material 2. Appendix 2. Online survey information sheet. Information sheet detailing the research and its conditions, sent to participants at the time of initial recruitment and presented to the UTS ethics committee. [file 12906_2026_5353_MOESM2_ESM.docx]

**Appendix 2: Online survey information sheet**

**FICHE D'INFORMATION POUR LES PARTICIPANT·E·S À L'ENQUÊTE EN LIGNE**

ONLINE SURVEY INFORMATION SHEET

**[APPROBATION COMMISSION ETHIQUE ETH22-7865]**

[UTS APPROVAL NUMBER ETH22-7865]

**PROJET HIPPOCRATE & SCIENCE (PHASE A) : CONSENSUS SUR LA THÉORIE DES TEMPÉRAMENTS ET SON APPLICATION EN NATUROPATHIE**

HIPPOCRATE & SCIENCE PROJECT (PHASE A) : CONSENSUS ON HIPPOCRATIC TEMPERAMENTS THEORY AND ITS APPLICATION IN NATUROPATHY

**QUI CONDUIT CETTE RECHERCHE?**

WHO IS CONDUCTING THIS RESEARCH?

**Ce projet de recherche est mené par l’équipe du Navi, pôle de recherche en santé intégrative (Suisse), en collaboration avec l’Université de Technologie de Sydney (UTS).**

**L’équipe de recherche est constituée de trois personnes principales :
Dr Amie Steel est chercheuse en chef du projet. Elle est chercheuse post-doctorale à l'UTS, et vice-présidente du registre australien des naturopathes et des herboristes [ARONAH] ; courriel** [**Amie.Steel@uts.edu.au**](mailto:Amie.Steel@uts.edu.au)**.**

**Anne Gimalac est co-responsable de la recherche. Elle est biologiste et responsable du Navi ; courriel** [**anne.gimalac@epsn.ch**](mailto:anne.gimalac@epsn.ch)

**Ludivine Colas est également co-responsable de la recherche. Elle est psychologue, naturopathe et chargée de recherche au Navi ; courriel** [**ludivine.colas@epsn.ch**](mailto:ludivine.colas@epsn.ch)

This research project is being conducted by the team at Navi, a research center for integrative health (Switzerland), in collaboration with the University of Technology Sydney (UTS).

The research team consists of three main individuals:

Dr. Amie Steel is the chief investigator on the project. She is a Post-doctoral Research Fellow UTS, Deputy-Chair of the Australian Register of Naturopaths and Herbalists [ARONAH]; email [Amie.Steel@uts.edu.au](mailto:Amie.Steel@uts.edu.au)

Anne Gimalac is the research co-investigator. She is a biologist and head of Navi; email [anne.gimalac@centre-navi.ch](mailto:anne.gimalac@epsn.ch)

Ludivine Colas is also co-investigator of the research. She is a psychologist, naturopath and research fellow at Navi; email [ludivine.colas@epsn.ch](mailto:ludivine.colas@epsn.ch)

**EN QUOI CONSISTE LA RECHERCHE?**

WHAT IS THE RESEARCH ABOUT?

**L'objectif de cette recherche/enquête en ligne est d’aboutir à un consensus qui reflète la réalité de la pratique et de l'enseignement de la théorie hippocratique dans la communauté naturopathique francophone. Cette étape permet de proposer une base conceptuelle scientifique commune aux naturopathes francophones et d’envisager la création d'un futur outil d'évaluation des tempéraments naturopathiques.**

The purpose of this research/online survey is to find a consensus that reflects the reality of the practice and teaching of Hippocratic theory in the French-speaking naturopathic community. This step allows us to propose a common scientific conceptual base for French-speaking naturopaths and to consider the creation of a future naturopathic temperament assessment tool.

**Vous avez été invité·e à participer car vous êtes praticien·ne et/ou enseignant·e en naturopathie dans un pays francophone et que vous travaillez d’une manière ou d’une autre avec la théorie des tempéraments d’Hippocrate comme outil d’évaluation du *terrain*.**

You have been invited to participate because you are a naturopathic practitioner and/or teacher in a French-speaking country and you work in one way or another with the Hippocratic temperament theory as a *“terrain”* (= evolutive constitution) assessment tool.

**FINANCEMENT**

FUNDING

**Les financements pour ce projet ont été obtenus de l’Ecole Professionnelle Supérieure de Naturopathie (EPSN Sàrl) basée en Suisse. Aucun conflit d'intérêt ou intérêt commercial n’est à déclarer.**

Funding for this project was obtained from the Swiss-based Professional High School of Naturopathy (EPSN Sàrl). No conflicts of interest or commercial interests are to be declared.

**QU’IMPLIQUE MA PARTICIPATION?**

WHAT DOES MY PARTICIPATION INVOLVE?

**La participation à cette étude se fait sur une base volontaire. Vous êtes entièrement libre de décider si vous voulez y participer ou non. Si vous décidez de participer, vous devrez répondre à 4 tours de questionnaire en ligne, répartis sur 4 à 6 mois. Vous pourrez y accéder en cliquant sur un lien qui vous sera envoyé par email tous les mois environ. Chaque questionnaire prendra approximativement 15 à 20 minutes à remplir.**

**Si vous décidez de ne pas participer à l’étude, cela n'affectera pas votre relation avec les chercheur·euse·s du Navi, l'Université de Technology de Sydney (UTS) ou l’institution qui vous a transmis l’annonce de cette étude (WNF, OMNES, …). Si vous souhaitez vous retirer de l'étude une fois qu'elle a commencé, vous pouvez le faire à tout moment sans avoir à donner de raison, en contactant** [anne.gimalac@centre-navi.ch](mailto:anne.gimalac@epsn.ch) **ou** [ludivine.colas@epsn.ch](mailto:ludivine.colas@epsn.ch)**. Vos précédentes réponses ne pourront alors pas être prises en compte dans les résultats de l’étude.**

**Sachez que cette étude implique un traitement anonyme des réponses et que votre nom ou prénom ne seront pas demandés. Seule votre adresse email sera retenue dans les données collectées à des buts de diffusion des questionnaires et de restitution des résultats de recherche.**

**Si vous décidez de quitter le projet de recherche, les informations personnelles déjà collectées (comme votre genre, votre pays de pratique ou votre institution d’affiliation) seront conservées afin de garantir que les résultats du projet de recherche puissent être mesurés correctement et pour se conformer à la loi.**

Participation in this study is voluntary. It is entirely up to you to decide whether or not you want to participate. If you decide to participate, you will be asked to complete 4 rounds of online questionnaire, spread over 4 to 6 months. You will be able to access them by clicking on a link that will be sent to you by email every month or so. Each survey will take approximately 15-20 minutes to complete.

If you decide not to participate in the study, this will not affect your relationship with the Navi researchers, the University of Technology Sydney (UTS) or the institution that sent you the announcement of this study (WNF, OMNES, ...). If you wish to withdraw from the study once it has started, you can do so at any time without giving a reason, by contacting anne.gimalac@epsn.ch or ludivine.colas@epsn.ch. Your previous responses will not be included in the study results.

Please note that this study involves anonymous processing of responses and that your name or first name will not be requested. Only your email address will be retained in the data collected for the purpose of disseminating the questionnaires and reporting the research results.

If you decide to leave the research project, personal information already collected (such as your gender, country of practice, or institution of affiliation) will be retained to ensure that the results of the research project can be properly measured and to comply with the law.

**QUELS SONT LES RISQUES/INCONVÉNIENTS ?**

ARE THERE ANY RISKS/INCONVENIENCE?

**Nous ne nous attendons pas à ce que ce questionnaire cause un préjudice ou une gêne quelconque. Les informations demandées touchent à votre expérience de l’évaluation du terrain des patient·e·s/client·e·s à l’aide de la théorie des tempéraments hippocratiques. Vous pouvez toujours répondre par le neutre si vous ne pouvez pas vous positionner en termes d’accord. Il n’y a pas de juste ou de faux et aucun jugement ne sera porté sur votre pratique ou vos compétences professionnelles. Si toutefois cette recherche occasionnait chez vous un inconfort ou une détresse, veuillez soit vous référer à votre médecin de famille, soit nous avertir pour que nous puissions vous soutenir adéquatement ou discuter d’une fin de participation.**

We don’t expect this questionnaire to cause any harm or discomfort. The information requested relates to your experience in assessing the patient’s “terrain” (= evolutive constitution) using Hippocratic temperament theory. You can always answer with the neutral option if you can't position yourself in terms of agreement. There is no right or wrong and no judgment will be made about your practice or professional skills. If, however, this research causes you discomfort or distress, please either refer to your family physician, notify us so that we can provide you with adequate support or discuss ending your participation.

**QU'ADVIENDRA-T-IL DES INFORMATIONS ME CONCERNANT ?**

WHAT WILL HAPPEN TO INFORMATION ABOUT ME?

**L'accès au questionnaire en ligne se fait via [détails du lien à venir]. La soumission des questionnaires en ligne est une indication de votre consentement.**

**Aucune information permettant d'identifier les participants ne sera collectée. Seule votre adresse email est nécessaire à la recherche dans un but de communication (envoie des questionnaires et restitution des résultats de recherche). Tous les paramètres de la plateforme d'enquête protégeront l'identité des participant·e·s.**

Access to the online questionnaire is via **[link details to follow**]. Submission of online questionnaires is an indication of your consent.

No personally identifiable information will be collected. Only your email address is required for research communication purposes (sending questionnaires and reporting research results). All settings on the survey platform will protect the identity of participants.

**Conformément aux lois australiennes et/ou néo-zélandaises sur la protection de la vie privée, vous avez le droit de demander l'accès aux informations vous concernant qui sont recueillies et stockées par l'équipe de recherche. Vous avez également le droit de demander que toute information avec laquelle vous n'êtes pas d'accord soit corrigée. Veuillez informer le membre de l'équipe de recherche dont le nom figure à la fin de ce document si vous souhaitez accéder à vos informations. Cela ne sera possible que si vous fournissez des informations personnelles susceptibles de vous identifier individuellement, ou d'être raisonnablement identifiables (par exemple, si des réponses en texte libre vous identifient ou vous ré-identifient de manière contextuelle).**

In accordance with relevant Australian and/or NSW Privacy laws, you have the right to request access to the information about you that is collected and stored by the research team. You also have the right to request that any information with which you disagree be corrected. Please inform the research team member named at the end of this document if you would like to access your information. This will only be possible if you provide personal information which may individually identify you, or be reasonably identifiable (e.g. if any open-text responses contextually identify or re-identify you).

**Il est prévu que les résultats de ce projet de recherche soient publiés et/ou présentés dans divers conférences. Les résultats de cette recherche peuvent également être partagés par le biais de bases de données scientifiques en libre accès (publiques), y compris des bases de données Internet. Cela permettra à d'autres chercheurs d'utiliser les données pour étudier d'autres questions de recherche importantes. Les résultats ainsi partagés sont toujours dépersonnalisés et ne comportent aucun moyen d’identifier les participant·e·s. Vos coordonnées personnelles seront stockées sur un Drive protégé par un mot de passe, auquel seul·e·s les chercheur·e·s auront accès.**

It is anticipated that the results of this research project will be published and/or presented in a variety of conferences. The results of this research may also be shared through open access (public) scientific databases, including internet databases. This will enable other researchers to use the data to investigate other important research questions. Results shared in this way will always be de-identified and have no way of identifying participants. Your personal contact details will be stored on the researcher’s password protected Drive which only the researchers have access to.

**QUE FAIRE SI J'AI DES QUESTIONS OU DES PRÉOCCUPATIONS ?**

WHAT IF I HAVE ANY QUERIES OR CONCERNS?

**Si vous avez des questions ou des préoccupations concernant la recherche et que vous pensez que nous pouvons vous aider, n'hésitez pas à nous contacter aux adresses suivantes :**

If you have any queries or concerns about the research that you think we can help you with, please feel free to contact us on :

**Navi**

**Biopôle, Bâtiment Alanine**

**Route de la Corniche 5**

**1066 Epalinges**

**Switzerland**

**Anne Gimalac Email:** [anne.gimalac@centre-navi.ch](mailto:anne.gimalac@epsn.ch) **Téléphone: +41 79 103 24 98**

**Ludivine Colas Email:** [ludivine.colas@epsn.ch](mailto:ludivine.colas@epsn.ch) **Téléphone: +33 6 84 03 03 19**

**Dr Amie Steel Email:** [Amie.Steel@uts.edu.au](mailto:Amie.Steel@uts.edu.au)

**Si vous souhaitez parler à quelqu'un qui n'est pas lié·e à la recherche, ou si vous avez des préoccupations ou des plaintes concernant un aspect quelconque de la conduite de cette recherche que vous souhaitez soulever indépendamment de l'équipe de recherche, veuillez contacter le Secrétariat d'éthique au +61 2 9514 2478, par courriel ou à l'adresse** [**Research.ethics@uts.edu.au**](mailto:Research.ethics@uts.edu.au)**, en indiquant le numéro de référence UTS HREC. Toute question soulevée sera traitée de manière confidentielle, fera l'objet d'une enquête et vous serez informé du résultat.**

If you would like to talk to someone who is not connected with the research, or if you have any concerns or complaints about any aspect of the conduct of this research that you wish to raise independently of the research team, please contact the Ethics Secretariat on +61 2 9514 2478 email or [Research.ethics@uts.edu.au](mailto:Research.ethics@uts.edu.au) and quote the UTS HREC reference number. Any matter raised will be treated confidentially, investigated and you will be informed of the outcome.
