# Supplementary material for: Establishing a consensus on the clinical assessment of Hippocratic temperaments in the French-speaking naturopathic community: a Delphi study
Source: BMC Complement Med Ther. 2026 Mar 29;26:173. doi: 10.1186/s12906-026-05353-y (PMC13151160; doi:10.1186/s12906-026-05353-y)
Supplement: Supplementary file 3 — Supplementary Material 3. Appendix 3. Selected texts from the literature review. A literature review of 23 books, scientific publications and professional content was carried out between June and August 2022. The full list of these references was provided to participants in the first survey round, informing them of the literary sources used to construct the questionnaires and inviting them to suggest additional references they considered important sources of knowledge on Hippocratic temperaments. [file 12906_2026_5353_MOESM3_ESM.docx]

**Appendix 3: Selected texts from the literature review**

1. Buckingham RM. Extraversion, neuroticism and the four temperaments of antiquity: an investigation of physiological reactivity. Personality and Individual Differences. 2002/01/19/ 2002;32(2):225-246. <https://doi.org/10.1016/S0191-8869(01)00020-4>
2. Carton P. Diagnostic et conduite des tempéraments. Librairie Le François: Paris; 1961.
3. Carton P. Traité de médecine, d'alimentation et d'hygiène naturistes. 2^nd^ ed. A. Maloine & Fils: Paris; 1924.
4. Dammeyer J, Zettler I. A Brief Historical Overview on Links Between Personality and Health. In: Personality and Disease. (Johansen C, ed.) Academic Press; 2018:1-16.
5. Garvelmann F. Konstitutionsmedizin. Bacopa-Verlag: Schiedlberg; 2018.
6. Gex M. Les classifications des tempéraments. Revue de Théologie et de Philosophie.1949; 37(152), 147-162.
7. Gunsburger N. Mon coach naturo : Mon programme sur mesure pour vivre en pleine santé. Eyrolles: Paris; 2017.
8. Howart E. Mood differences between the four Galen personality types: choleric, sanguine, phlegmatic, melancholic. Personality and Individual Differences. 1988/01/01/ 1988;9(1):173-175. <https://doi.org/10.1016/0191-8869(88)90044-X>
9. Hutter L. Diagnostic humoral de la langue. Nhk Institut für integrative Naturheilkunde; 2017.
10. Jouanna J. La théorie des quatre humeurs et des quatre tempéraments dans la tradition latine (Vindicien, Pseudo-Soranos) et une source grecque retrouvée. Revue des Études Grecques. 2005;118(1):138-167. <https://www.persee.fr/doc/reg_0035-2039_2005_num_118_1_4609>
11. Kieffer D. Guide personnel des bilans de santé : Encyclopédie naturopathique des tests morphologiques, psychologiques et biologiques de terrain. new ed. Grancher: Escalquens; 2004.
12. King H, Dasen V. La médecine dans l’antiquité grecque et romaine. Editions BHMS: Lausanne; 2008.
13. Léaud-Zachoval D. Quatre clefs pour la santé : Lymphatique, sanguin, bilieux, nerveux, qui suis-je vraiment ? Editions Médicis: Paris; 2017.
14. Léaud-Zachoval D. Voyage au centre de la naturopathie. Hippocrate. 2021;2(5):17-25.
15. Osborn D. Greek medicine. Updated 2027. Available from: http://greekmedicine.net/ [last accessed July 27, 2024].
16. Openpsychometrics. OSPP Four Temperaments Test. Openpsychometrics Website. Published March 19, 2019, updated 1 June 2023. Available from <https://openpsychometrics.org/tests/O4TS/> [last accessed July 27, 2024].
17. Raimann C, Ganz C, Garvelmann F, et al. Grundlagen der Traditionellen Europäischen Naturheilkunde TEN. BACOPA: Schiedlberg; 2017.
18. Rolfe R. The Four Temperaments. Marlowe & Compagny: New York; 2002.
19. Ruch W. Pavlov's types of nervous system, Eysenck's typology and the Hippocrates-Galen temperaments: An empirical examination of the asserted correspondence of three temperament typologies. Personality and Individual Differences. 1992/12/01/ 1992;13(12):1259-1271. <https://doi.org/10.1016/0191-8869(92)90168-O>
20. Salmani Nodoushan MA. Temperament as an indicator of language achievement. Intern J Language Studies. 10/01 2011;5:33-52.
21. Ternisien L. Naturopathie, le guide saison par saison. J’ai lu: Paris; 2020.
22. Vanopdenbosch Y. Les tempéraments : Outil de connaissance de soi et des autres. Amyris: Bruxelles; 2012.
23. Voutsinas D. Tempérament, constitution, caractère. Bulletin de psychologie. 1961;15(197), 25-40. [ttps://www.persee.fr/doc/bupsy_0007-4403_1961_num_15_197_8581](https://www.persee.fr/doc/bupsy_0007-4403_1961_num_15_197_8581)
